# Systematic review and meta-analysis: analysis of variables influencing the interpretation of clinical trial results in NAFLD

**DOI:** 10.1007/s00535-022-01860-0

**Published:** 2022-03-24

**Authors:** Javier Ampuero, Rocío Gallego-Durán, Douglas Maya-Miles, Rocío Montero, Sheila Gato, Ángela Rojas, Antonio Gil, Rocío Muñoz, Manuel Romero-Gómez

**Affiliations:** 1 Digestive Disease Department, Virgen del Rocio University Hospital, Avenida Manuel Siurot s/n, 41013 Sevilla, Spain; 2 SeLiver Group, Instituto de Biomedicina de Sevilla/CSIC, Universidad de Sevilla, Sevilla, Spain; 3 Hepatic and Digestive Diseases Networking Biomedical Research Centre (CIBERehd), Madrid, Spain

**Keywords:** NAFLD, Fibrosis, NASH, Placebo, Drug

## Abstract

**Background:**

NAFLD clinical trials have shown suboptimal results, particularly for liver fibrosis, despite the robust preclinical drug development. We aimed to assess the histological response after the experimental treatment versus placebo by carrying out a meta-analysis of NAFLD clinical trials.

**Methods:**

After a systematic review of NAFLD clinical trials to May 2021, applying strict selection criteria, the following primary outcomes were observed: (a) NASH resolution, with no worsening of fibrosis when available; (b) fibrosis improvement  ≥ 1 stage, with no worsening of NAS when available; (c) worsening of NAS; (d) worsening of liver fibrosis  ≥ 1 stage, including the progression to cirrhosis on histopathology. Other histological, clinical, and biochemical outcomes were considered secondary endpoints. Heterogeneity was explored by subgroup and sensitivity analyses, and univariable meta-regression.

**Results:**

Twenty-seven randomized clinical trials were included. The pooled efficacy for NASH resolution receiving experimental therapy was 19% (95%CI 15–23; *I*^2^ 96.2%) compared with placebo 10% (95%CI 7–12; *I*^2^ 85.8%) (OR 1.66 (95%CI 1.24–2.21); *I*^2^ 57.8%), while it was 26% (95%CI 22–29); *I*^2^ 90%)) versus 18% (95%CI 15–21; *I*^2^ 59%)) for fibrosis improvement (OR 1.34 (95%CI 1.13–1.58); *I*^2^ 25.4%). For these outcomes, the therapy showed higher efficacy in trials longer than 48 weeks, with  < 60% of diabetic population, and when it targeted FXR, PPAR, and antidiabetic mechanisms, and with a NAS  < 5 for NASH resolution. Also, NASH (OR 0.57 (95%CI 0.39–0.84); *I*^2^ 67%) and fibrosis worsening (OR 0.65 (95%CI 0.46–0.92); *I*^2^ 61.9%) were prevented with the therapy.

**Conclusion:**

This meta-analysis provides information about the efficacy of the therapy versus placebo by comparing different and combined trial outcomes such as NASH resolution, fibrosis improvement, and NAS and fibrosis worsening. Changes in the experimental design and selection criteria of the clinical trials might be suitable to increase the efficacy.

**Supplementary Information:**

The online version contains supplementary material available at 10.1007/s00535-022-01860-0.

## Introduction

Within the past decade, the individual contribution of distinct etiologies to the burden of liver diseases has switched from viral hepatitis towards non-alcoholic fatty liver disease (NAFLD) [1]. NAFLD constitutes a complex metabolic disorder that manifests with fat accumulation in the cytoplasm of the hepatocyte in the absence of significant alcohol consumption or other causes of liver diseases [2]. Moreover, NAFLD severity ranges from simple steatosis to non-alcoholic steatohepatitis (NASH), usually accompanied by different stages of liver fibrosis, ballooning, and overall chronic inflammation status. This disease has surprisingly become a leading cause of liver cirrhosis and hepatocellular carcinoma worldwide. The risk of liver-related mortality exponentially grows with an increase in fibrosis stages [3]; therefore, the diagnosis of liver fibrosis is one of the first steps when stratifying patients prior to the inclusion in clinical trials. Besides, NAFLD plays a catalytic role in the development of metabolic comorbidities in these multimorbid patients. Significant fibrosis but not simple steatosis or NASH predicts type 2 diabetes mellitus (T2DM) and arterial hypertension in these patients [4].

Despite its enormous prevalence, no regulatory-approved therapeutic option has been authorized yet [5]; therefore. the cornerstone of NAFLD management still relies on lifestyle interventions [6]. In the context of clinical trials, the identification of patients at risk of suffering from liver-related and non-liver-related complications is tough. Besides, identifying the most appropriate therapy in NAFLD patients still remains a challenge. Recent studies have pointed out that the ideal treatment should address liver fibrosis and NASH in a joint fashion. To date, clinical trials have fallen short when testing the efficacy of novel molecular targets due to changes in some uncontrolled variables, such as dysmetabolic comorbidities and/or daily habits, since they are not reported nor adequately represented [7]. Considering hepatic fibrosis as a crucial factor of clinical prognosis and reinforcing the role of inflammation and disease activity as key players in the maintenance of chronicity in this disease, both factors should be taken into account as primary clinical trial endpoints.

Data obtained from NAFLD clinical trials have shown suboptimal results, particularly for liver fibrosis, despite the robust preclinical development of the therapies. Therefore, in this setting, we carried out a meta-analysis to assess the histological response after the experimental treatment versus placebo (including NASH resolution and fibrosis improvement  ≥ 1 stage) and, as a main novelty, the clinical benefits in delaying disease progression.

## Methods

### Study identification and selection

We conducted our review according to the PRISMA reporting guideline for systematic reviews [8]. One of the reviewers (JA) with experience in database searches designed the search strategy, which was subsequently revised by other three investigators (RG, DM, AR). They independently searched MEDLINE (using PUBMED as the search engine), EMBASE, and Cochrane databases and collected all results separately. Disagreements between them were resolved by a third investigator (MRG) or by consensus. Databases were used to identify suitable studies that were published up to 1 May 2021. MeSH terms and keywords were used, and the search terms were as follows: NAFLD, MAFLD, NASH, non-alcoholic fatty liver disease, non-alcoholic steatohepatitis, fatty liver, liver fat, steatosis, clinical trial, treatment, therapy, drug, and a combination of those MeSH terms by using the appropriate Boolean logic. The searches were limited to English-language publications with human subjects. A manual search was conducted using the references listed in the original articles and review articles retrieved. Only fully published articles and oral presentations subjected to the same assessment as regular articles (AASLD and EASL meetings) were considered, so abstracts and posters were not considered. The inclusion criteria were as follows: (a) randomized clinical trial; (b) placebo-controlled clinical trial; (c) Phase II and Phase III clinical trial; (d) paired biopsy; (e) adults (≥ 18 years old). The exclusion criteria were as follows: (a) duplicate reports; (b) case reports, comments, and letters to the editors; (c) systematic reviews or meta-analyses; (d) botanical products, herbal medicines, or antioxidants; (e) lifestyle intervention.

### Data extraction and quality assessment

The following data were extracted: author, year, population selection criteria, sample size, experimental drug, histological endpoint (NASH, NAFLD Activity Score (NAS), fibrosis stage, steatosis, lobular inflammation, ballooning), biochemical response (AST, ALT), age, sex, body mass index (BMI), T2DM. When the same population was published in several journals, we retained only the most informative article or the most complete study to avoid duplication. We also asked the investigators for additional information, and if we received no answer, “unreported” items were treated as “unclear” or “not available”. On the other hand, four investigators (AG, SG, RM, RM) independently assessed the quality of the studies using the “Quality in Prognostic Studies (QUIPS)” tool [9].

### Outcome measures

Given that the ultimate goal of NASH treatment is to slow the progress of, halt, or reverse disease progression and improve clinical outcomes, we selected the following the histological response after experimental treatment or placebo as the primary outcome: (a) NASH resolution, with no worsening of fibrosis when available; (b) fibrosis improvement ≥ 1 stage, with no worsening of NAS when available. On the other hand, as a clinical benefit can be verified by demonstrating superiority to placebo in delaying disease progression, we additionally considered: (a) worsening of NAS; (b) worsening of liver fibrosis ≥ 1 stage, including the progression to cirrhosis on histopathology. In addition, other histological outcomes were assessed as secondary endpointsasfollows: (a) NAS improvement > 2 points, irrespective of fibrosis improvement; (b) improvement of steatosis, lobular inflammation, and ballooning. Also, the occurrence of cirrhosis complications was analyzed. Finally, the biochemical response (ALT, AST) was also assessed.

### Statistical analysis

We used STATA version 16 (Stata Corp; College Station, TX). All statistical tests were two-sided, with *P*-values ≤ 0.05 denoting statistical significance. Confidence intervals (CIs) of individual studies were determined from the available data. For the dichotomous variables, the effect denotes odds ratio (OR) and corresponding 95% CIs, while we used the difference in means to specifically provide measures of the absolute difference between the mean values of the explored variables. To estimate the pooled prevalence, the prevalence rates were combined in a random-effects meta-analysis.

The assumption of heterogeneity was tested for each planned analysis using the Cochran-Q heterogeneity and I^2^ statistics (significant heterogeneity according to I^2^ value > 50%) [10]. The random-effects model was applied to pool results from studies. We planned a priori subgroup analyses according to the following criteria: trials with cirrhotic versus non-cirrhotic population (three studies included 100% of cirrhotic patients, while other two included 50% and one 11%; however, they were considered as a cirrhotic population because separated information was not available), trials with ≥ 60% versus  < 60% of diabetic population, trials with mean NAS less than 5 versus 5 or greater, treatment duration 48 weeks or less versus greater than 48 weeks, and therapeutic class (Supplementary Table 1). Additionally, significant heterogeneity for primary outcomes was explored by univariable meta-regression, and a sensitivity analysis was performed to determine if there was any undue influence exerted by a single study on the results of the combined studies [11, 12]. Finally, the potential publication bias was assessed by Egger’s test and graphically by a funnel plot when there was an adequate number of studies (> 10 studies).

## Results

### Eligible study characteristics and quality assessment

The flowchart diagram details the article selection process for this meta-analysis (Supplementary Fig. 1), which ended with 27 studies included. The characteristics of the eligible studies are listed in Table 1. Supplementary Table 2 shows the quality assessment of the clinical trials by QUIPS.

**Table 1:** Characteristics of the studies included in the meta-analysis

Drug	First author	Year	Phase	Population	Intervention	Duration	Histological endpoints
						(weeks)	
Aldafermin [14]	Stephen A. Harrison	2021	IIb	*s* = 78 NAS (mean): 5.6 Fibrosis (%): F2 56, F3 44 Diabetes (%): 61.5% BMI (mean): 36.1 kg/m^2^	Aldafermin 1 mg (*n* = 53) Placebo (*n* = 25)	24	NASH resolution & no worsening of fibrosis Improvement > 2 NAS points & no worsening of fibrosis Fibrosis improvement ≥ 1 stage & no worsening of NAS Improvement of individual components of NAS
Aramchol [35]	Vlad Ratziu	2020	IIb	*N* = 247 NAS (mean): 5.12 Fibrosis (%): F1 40, F2 20, F3 40 Diabetes (%): All diabetic or prediabetic BMI (mean): 32.7 kg/m^2^	Aramchol 400 mg (*n* = 101) Aramchol 600 mg (*n* = 98) Placebo (*n* = 48)	52	NASH resolution & no worsening of fibrosis Fibrosis improvement ≥ 1 stage & no worsening of NAS Progression to cirrhosis
Belapectin [15]	Naga Chalasani	2020	IIb	*N* = 162 NAS (mean): 4.2 Fibrosis (%): F4 100 Diabetes (%): 60.5 BMI (mean): 34.9 kg/m^2^	Belapectin 2 mg/kg (*n* = 54) Belapectin 8 mg/kg (*n* = 54) Placebo (*n* = 54)	52	Fibrosis improvement ≥ 1 stage Cirrhosis complications
Cenicriviroc [16]	Scott L. Friedman	2018	IIb	*N* = 289 NAS (mean): 5.3 Fibrosis (%): F1 33, F2 28, F3 38 Diabetes (%): 50.5 BMI (mean): 33.9 kg/m2	CVC 150 mg (*n* = 145) Placebo (*n* = 144)	52	NASH resolution & no worsening of fibrosis Improvement > 2 NAS points & no worsening of fibrosis Fibrosis improvement ≥ 1 stage & no worsening of NAS Improvement of individual components of NAS Fibrosis worsening ≥ 1 stage Progression to cirrhosis Worsening of individual components of NAS
Cilofexor-Fircostotat [17]	Rohit Loomba	2020	IIb	*N* = 392 NAS (mean): N/A (90% of population with NAS > 5) Fibrosis (%): F3 50, F4 50 Diabetes (%): 72 BMI (mean): 33 kg/m^2^	Selonsertib 18 mg (*n* = 39) Firsocostat 20 mg (*n *= 40) Cilofexor 30 mg (*n* = 40) Selonsertib + Cilofexor (*n* = 77) Selonsertib + Firsocostat (*n* = 79) Firsocostat + Cilofexor (*n* = 78) Placebo (*n* = 39)	48	NASH resolution & no worsening of fibrosis Improvement > 2 NAS points Fibrosis improvement ≥ 1 stage & no worsening of NAS Improvement of individual components of NAS Progression to cirrhosis
Efruxifermin [18]	Stephen A. Harrison	2021	IIa	*N* = 42 No cirrhosis NAS (mean): 5.4 Fibrosis (%): F1 36, F2 32, F3 32 Diabetes (%): 51.5 BMI (mean): 37.6 kg/m2 *N* = 17 Cirrhosis (Cohort C) NAS (mean): N/A Fibrosis (%): F4 100 Diabetes (%): N/A BMI (mean): N/A	No cirrhosis Efruxifermin 28 mg (*n* = 13) Efruxifermin 50 mg (*n* = 13) Efruxifermin 70 mg (*n* = 14) Placebo (*n* = 2) Cirrhosis Efruxifermin 50 mg (*n* = 12) Placebo (*n* = 5)	16	No cirrhosis NASH resolution & no worsening of fibrosis Improvement > 2 NAS points & no worsening of fibrosis Fibrosis improvement ≥ 1 stage & no worsening of NAS Improvement of individual components of NAS Fibrosis worsening ≥ 1 stage Cirrhosis NASH resolution & no worsening of fibrosis Fibrosis improvement ≥ 1 stage & no worsening of NAS
Elafibranor [19]	Vlad Ratziu	2016	IIb	*N* = 276 NAS (mean): 5 Fibrosis (%): F0 15, F1 36, F2 26, F3 23 Diabetes (%): 39 BMI (mean): 31.2 kg/m^2^	Elafibranor 80 mg (*n* = 93) Elafibranor 120 mg (*n* = 91) Placebo (*n* = 92)	52	NASH resolution & no worsening of fibrosis Improvement > 2 NAS points
Elafibranor [36]	Stephen A. Harrison	2020	III	*N* = 1070 NAS (mean): 5.7 Fibrosis (%): F2 47, F3 53 Diabetes (%): 49.6 BMI (mean): 33.9 kg/m^2^	Elafibranor 120 mg (*n* = 717) Placebo (*n* = 353)	72	NASH resolution & no worsening of fibrosis Fibrosis improvement ≥ 1 stage & no worsening of NAS
Emricasan [20]	Stephen A. Harrison	2020	IIb	*N* = 318 NAS (mean): 5.5 Fibrosis (%): F1 21, F2 43, F3 36 Diabetes (%): 50.6 BMI (mean): 34 kg/m^2^	Emricasan 50 mg (*n* = 106) Emricasan 5 mg (*n* = 107) Placebo (*n* = 105)	72	NASH resolution & no worsening of fibrosis Improvement > 2 NAS points Fibrosis improvement ≥ 1 stage & no worsening of NAS Improvement of individual components of NAS NAS worsening Fibrosis worsening ≥ 1 stage Worsening of individual components of NAS
Lanifibranor [37]	Sven Francque	2021	IIb	*N* = 247 NAS (mean): 5.9 Fibrosis (%): F1 24, F2–F3 76 Diabetes (%): 42 BMI (mean): 32.9 kg/m^2^	Lanifibranor 800 mg (*n* = 83) Lanifibranor 1200 mg (*n* = 83) Placebo (*n* = 81)	24	NASH resolution & no worsening of fibrosis Improvement > 2 NAS points & no worsening of fibrosis Fibrosis improvement ≥ 1 stage & no worsening of NAS Improvement of individual components of NAS Fibrosis worsening ≥ 1 stage
Liraglutide [21]	Matthew James Armstrong	2016	IIb	*N* = 52 NAS (mean): 4.9 Fibrosis (%): F0–F2 49, F3 40, F4 11 Diabetes (%): 32.7 BMI (mean): 36 kg/m^2^	Liraglutide 1.8 mg (*n* = 26) Placebo (*n* = 26)	48	NASH resolution Improvement > 2 NAS points Fibrosis improvement ≥ 1 stage Improvement of individual components of NAS NAS worsening Fibrosis worsening ≥ 1 stage
MSDC-0602 K [32]	Stephen A. Harrison	2020	IIb	*N* = 392 NAS (mean): 5.3 Fibrosis (%): F1 38, F2 16, F3 45 Diabetes (%): 52.3 BMI (mean): 35.2 kg/m^2^	MSDC-0602 K 62.5 mg (*n* = 99) MSDC-0602 K 125 mg (*n* = 98) MSDC-0602 K 250 mg (*n* = 101) Placebo (*n* = 94)	52	NASH resolution Improvement > 2 NAS points & no worsening of fibrosis Fibrosis improvement ≥ 1 stage & no worsening of NAS
Obeticholic acid [22]	Brent A Neuschwander-Tetri	2015	II	*N* = 283 NAS (mean): 5.2 Fibrosis (%): F0–F1 29, F2 52, F3 22, F4 1 Diabetes (%): 53 BMI (mean): 34.5 kg/m^2^	Obeticholic acid 25 mg (*n* = 141) Placebo (*n* = 142)	72	NASH resolution Improvement > 2 NAS points & no worsening of fibrosis Fibrosis improvement ≥ 1 stage Improvement of individual components of NAS NAS worsening Fibrosis worsening ≥ 1 stage Worsening of individual components of NAS
Obeticholic acid [23]	Zobair M Younossi	2019	III	*N* = 931 NAS (mean): N/A (70% of the population with NAS > 6) Fibrosis (%): F2 44, F3 56 Diabetes (%): 54 BMI: N/A (mean body weight: 95 kgs)	Obeticholic acid 10 mg (*n* = 312) Obeticholic acid 25 mg (*n* = 308) Placebo (*n* = 311)	72	NASH resolution & no worsening of fibrosis Improvement > 2 NAS points & no worsening of fibrosis Fibrosis improvement ≥ 1 stage & no worsening of NAS Improvement of individual components of NAS Fibrosis worsening ≥ 1 stage
Pioglitazone [24]	Arun J. Sanyal	2010	IIb	*N* = 247 NAS (mean): 4.9 Fibrosis (%): F0 17, F1–F2 63, F3 18, F4 2 Diabetes (%): 0 BMI (mean): 34 kg/m^2^	Pioglitazone 30 mg (*n* = 80) Vitamin E 800 IU (*n* = 84) Placebo (*n *= 83)	96	NASH resolution Improvement > 2 NAS points & no worsening of fibrosis Fibrosis improvement ≥ 1 stage Improvement of individual components of NAS NAS worsening Fibrosis worsening ≥ 1 stage Worsening of individual components of NAS
Pioglitazone [25]	Kenneth Cusi	2016	IIb	*N* = 101 NAS (mean): 4.5 Fibrosis (%): N/A (mean fibrosis stage: 1) Diabetes (%): 51.4 BMI (mean): 34.4 kg/m2	Pioglitazone 45 mg (*n* = 50) Placebo (*n *= 51)	72	NASH resolution Improvement > 2 NAS points & no worsening of fibrosis Fibrosis improvement ≥ 1 stage Improvement of individual components of NAS
Resmetirom [26]	Stephen A Harrison	2019	IIb	*N* = 125 NAS (mean): 4.9 Fibrosis (%): F0 2, F1 53, F2 25, F3 20 Diabetes (%): 39.2 BMI (mean): 35.1 kg/m^2^	Resmetiron 80 mg (*n *= 84) Placebo (*n* = 41)	36	NASH resolution & no worsening of fibrosis Improvement > 2 NAS points Fibrosis improvement ≥ 1 stage & no worsening of NAS
Seladelpar [34]	Stephen A. Harrison	2020	II	*N* = 171 NAS (mean): 5.2 Fibrosis (%): F1 16, F2–F3 84 Diabetes (%): 48.5 BMI (mean): 36.1 kg/m2	Seladelpar 10 mg (*n* = 50) Seladelpar 20 mg (*n* = 47) Seladelpar 50 mg (*n* = 48) Placebo (*n* = 26)	52	NASH resolution & no worsening of fibrosis Fibrosis improvement ≥ 1 stage
Selonsertib [27]	Rohit Loomba	2018	IIb	*N* = 72 NAS (mean): N/A (70% of the population with NAS > 6) Fibrosis (%): F2 35, F3 65 Diabetes (%): 70.8 BMI (mean): 34 kg/m2	Selonsertib 18 mg + Simtuzumab 125 mg (*n* = 32) Selonsertib 6 mg + Simtuzumab 125 mg (*n* = 30) Simtuzumab 125 mg (*n* = 10)	24	Improvement > 2 NAS points Fibrosis improvement ≥ 1 stage Improvement of individual components of NAS Fibrosis worsening ≥ 1 stage Progression to cirrhosis
Selonsertib [28]	Stephen A. Harrison	2020	III	STELLAR 3: N = 802 NAS (mean): N/A (80% of the population with NAS > 5) Fibrosis (%): F3 100 Diabetes (%): 70.2 BMI (mean): 32.3 kg/m^2^ STELLAR 4: N = 877 NAS (mean): N/A (80% of the population with NAS > 5) Fibrosis (%): F4 100 Diabetes (%): 76.9 BMI (mean): 33 kg/m^2^	STELLAR-3 Selonsertib 18 mg (*n* = 322) Selonsertib 6 mg (*n* = 321) Placebo (*n* = 159) STELLAR-4 Selonsertib 18 mg (*n* = 354) Selonsertib 6 mg (*n* = 351) Placebo (*n *= 172)	48	STELLAR-3 NASH resolution & no worsening of fibrosis Fibrosis improvement ≥ 1 stage & no worsening of NAS Progression to cirrhosis STELLAR-4 NASH resolution & no worsening of fibrosis Fibrosis improvement ≥ 1 stage & no worsening of NAS Cirrhosis complications
Semaglutide [29]	P.N. Newsome	2021	IIb	*N* = 320 NAS (mean): 4.9 Fibrosis (%): F1 28, F2 23, F3 49 Diabetes (%): 62.2 BMI (mean): 35.7 kg/m^2^	Semaglutide 0.1 mg (*n* = 80) Semaglutide 0.2 mg (*n* = 78) Semaglutide 0.4 mg (*n* = 82) Placebo (*n* = 80)	72	NASH resolution & no worsening of fibrosis Fibrosis improvement ≥ 1 stage & no worsening of NAS Improvement of individual components of NAS Fibrosis worsening ≥ 1 stage Progression to cirrhosis Worsening of individual components of NAS
Simtuzumab [30]	Stephen A. Harrison	2018	IIb	*N* = 219 Bridging fibrosis: NAS (mean): 4.9 Fibrosis (%): F3 100 Diabetes (%): 67.1 BMI (mean): 33.7 kg/m^2^ *N* = 258 Cirrhosis: NAS (mean): 3.8 Fibrosis (%): F4 100 Diabetes (%): 69.4 BMI (mean): 33.5 kg/m^2^	Bridging fibrosis: SIM 75 mg (*n* = 71) SIM 125 mg (*n* = 74) Placebo (*n* = 74) Cirrhosis: SIM 200 mg (*n* = 87) SIM 700 mg (*n* = 86) Placebo (*n *= 85)	96	Bridging fibrosis: NASH resolution Improvement > 2 NAS points Fibrosis improvement ≥ 1 stage Progression to cirrhosis Cirrhosis: NASH resolution Improvement > 2 NAS points Fibrosis improvement ≥ 1 stage Cirrhosis complications
Tropifexor [33]	Kathryn J. Lucas	2020	IIb	*N* = 152 NAS (mean): 6 Fibrosis (%): F2 41, F3 59 Diabetes (%): 80.2 BMI (mean): 34.7 kg/m^2^	Tropifexor 140 mcg (*n* = 50) Tropifexor 200 mcg (*n* = 51) Placebo (*n* = 51)	48	NASH resolution & no worsening of fibrosis Fibrosis improvement ≥ 1 stage & no worsening of NAS Fibrosis worsening ≥ 1 stage
Volixibat [31]	Philip N. Newsome	2020	II	*N* = 197 NAS (mean): 5.2 Fibrosis (%): F0 17, F1 39, F2 13, F3 31 Diabetes (%): 43.4 BMI (mean): 34.5 kg/m^2^	Volixibat 5 mg (*n* = 49) Volixibat 10 mg (*n* = 50) Volixibat 20 mg (*n* = 49) Placebo (*n* = 49)	48	NASH resolution & no worsening of fibrosis Improvement > 2 NAS points & no worsening of fibrosis Fibrosis improvement ≥ 1 stage

### Data analyses about NASH

**Fig. 1:**
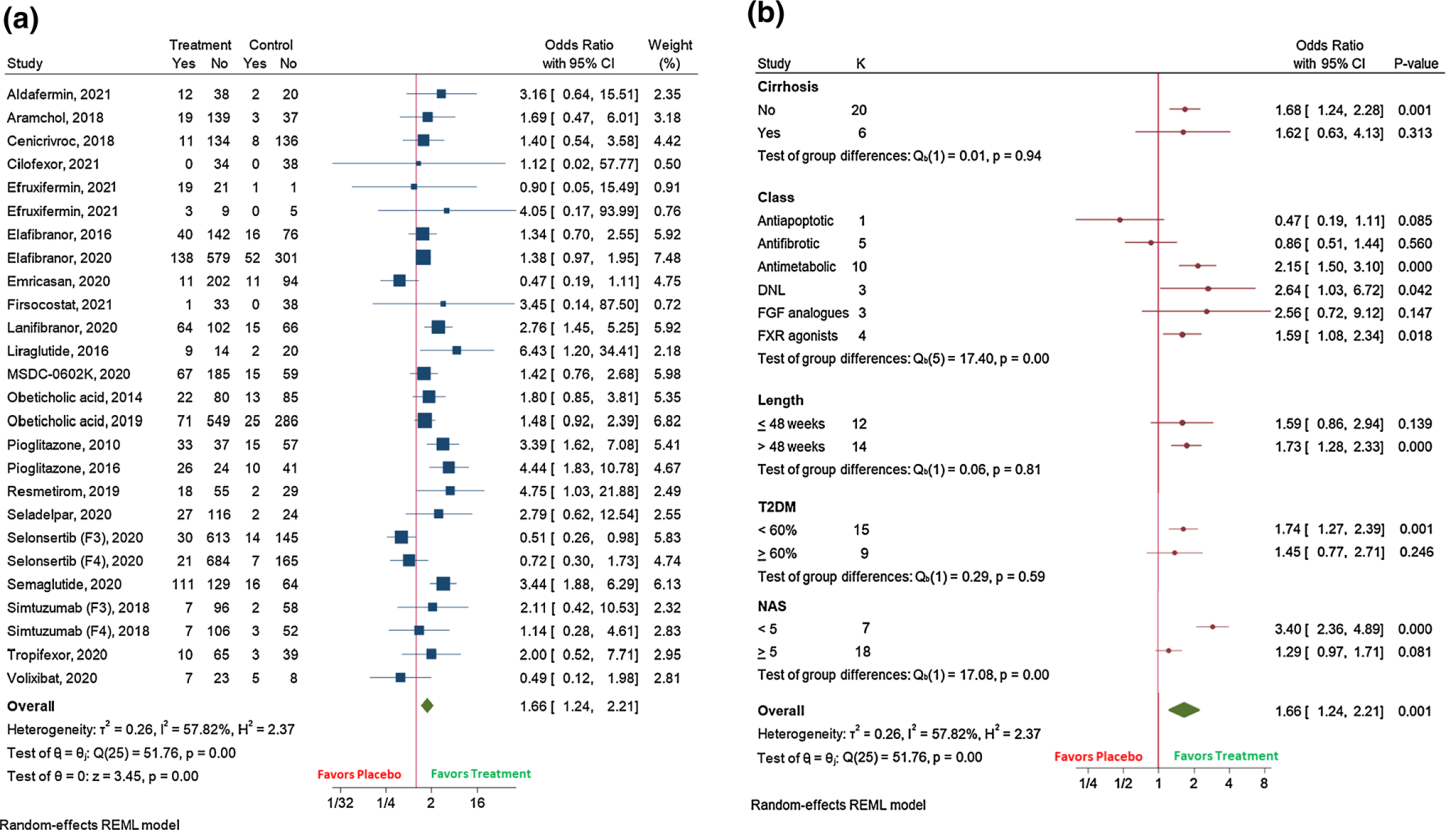
HYPERLINK "sps:id::fig1||locator::gr1||MediaObject::0" The effect of the experimental drug on:** (A)** NASH resolution; (**B)** NASH resolution, according to subgroup analyses

NASH resolution was assessed by 26 clinical trials (*N* = 7239 patients). The pooled efficacy for NASH resolution obtained by patients treated with any experimental drug was 19% (95%CI 15–23; *I*^2^ 96.2%) when compared with placebo 10% (95%CI 7–12; *I*^2^ 85.8%) (Supplementary Fig. 2). The treatment difference between receiving a therapy placebo was higher considering the studies evaluating additionally the lack of worsening of fibrosis (*N* = 17) (OR 2.32 (95%CI 1.67–3.23); *I*^2^ 4.9%) than considering the total of studies (*N* = 26) (OR 1.66 (95%CI 1.24–2.21); *I*^2^ 57.8%) (Fig. 1a). The subgroup analysis showed that NASH resolution was more difficult to achieve in cirrhotic in comparison with non-cirrhotic patients for both experimental therapy [(4% (95%CI 1–8; *I*^2^ 80.1%) versus 22% (95%CI 17–28; *I*^2^ 95.7%))] and placebo [(2% (95%CI 0–4; *I*^2^ 48%) versus 12% (95%CI 9–14; *I*^2^ 65.8%))] (Supplementary Fig. 2). In addition, the experimental drug showed higher efficacy in clinical trials with a mean NAS < 5 versus NAS ≥ 5, with a duration > 48 versus  ≤ 48 weeks, with less than 60% of diabetic population, and when it was based on antimetabolic mechanisms, targeting de novo lipogenesis (DNL) and FXR agonist (Fig. 1).

Improvement of NAS by  ≥ 2 points is another typical endpoint of clinical trials. Up to 19 studies assessed this outcome (*N* = 3798 patients). Overall, receiving an experimental treatment increased the likelihood of achieving this outcome (37% (95%CI 0.28–0.46; *I*^2^ 96.1%)) compared to placebo (23% (95%CI 0.16–0.30; *I*^2^ 89%)) (OR 1.72 (95%CI 1.23–2.41); *I*^2^ 71%) (Supplementary Fig. 3). Similar to NASH resolution, the improvement of NAS ≥ 2 points was higher in non-cirrhotic than in cirrhotic patients for the experimental (40% (95%CI 30–51; *I*^2^ 96.8%) and 24% (95%CI 14–34; *I*^2^ 57.4%), respectively) and placebo arms (26% (95%CI 18–34; *I*^2^ 90.3%) and 13% (95%CI 7–20; *I*^2^ 31.8%), respectively) (Supplementary Fig. 4, b).

On the other hand, NASH worsening was assessed in four clinical trials (*N* = 695). Patients treated with experimental therapy significantly displayed a lower rate of NASH worsening (14% (95%CI 5–23); *I*^2^ 83.2%) than those taking placebo (25% (95%CI 20–30); *I*^2^ 0%)) (Supplementary Fig. 5a,b), thus showing a protective effect of the medication (OR 0.57 (95%CI 0.39–0.84); *I*^2^ 67%) (Supplementary Fig. 6).

### Data analysis about fibrosis

**Fig. 2:**
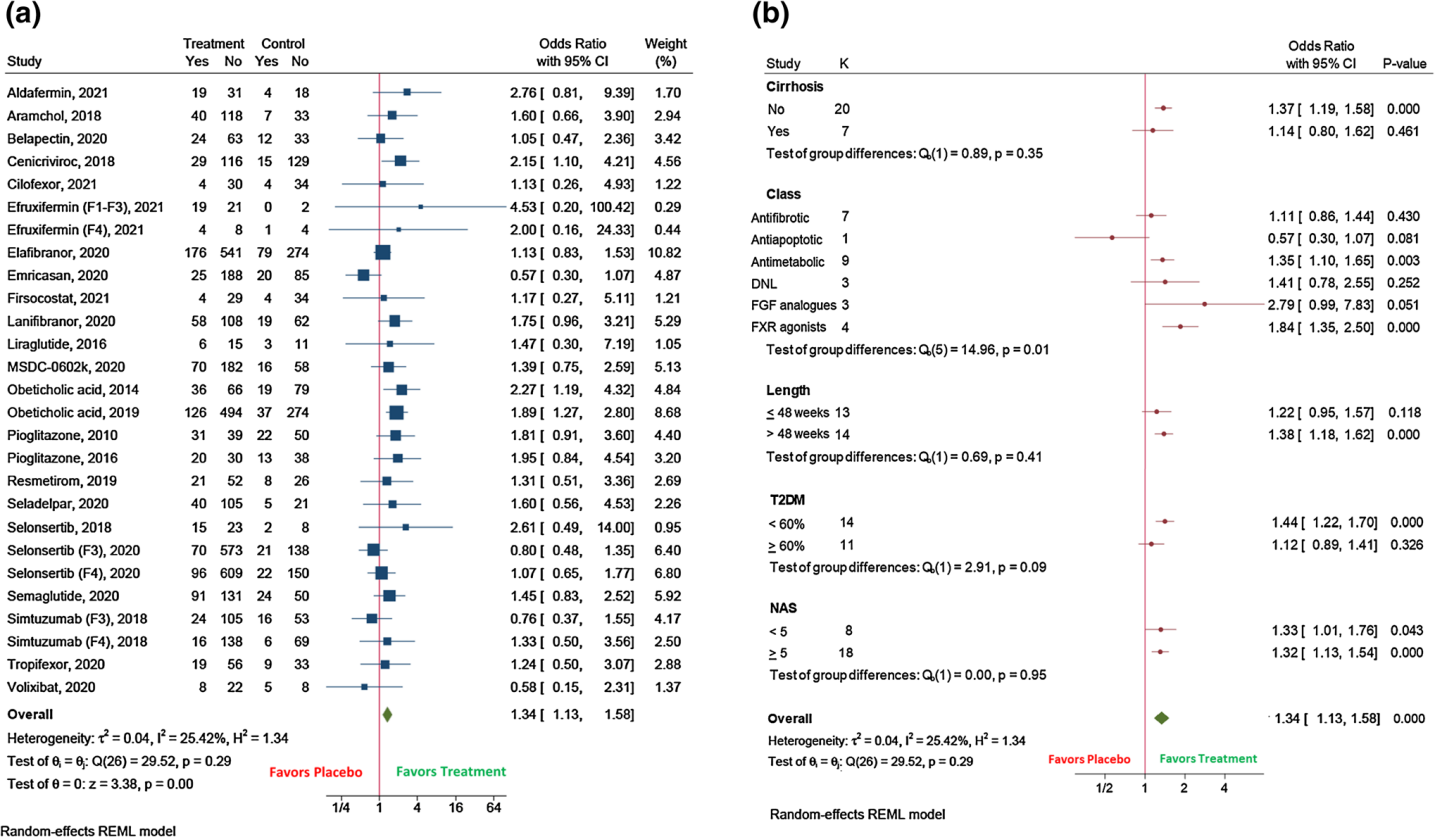
The effect of the experimental drug on: **A** Fibrosis improvement ≥ 1 stage; **B** Fibrosis improvement ≥ 1 stage, according to subgroup analyses

Fibrosis improvement  ≥ 1 stage was assessed by 27 clinical trials (*N* = 7151 patients). This analysis proved that the experimental therapy was superior, achieving 26% (95%CI 22–29); *I*^2^ 90%)) of this outcome versus 18% (95%CI 15–21; *I*^2^ 59%)) with placebo. The beneficial effect of the drug was similar in the studies that additionally assessed no worsening of NASH (*N* = 16) (OR 1.30 (95%CI 1.12–1.51); *I*^2^ 25.8%) in comparison with the total of the studies (*N* = 27) (OR 1.34 (95%CI 1.13–1.58); *I*^2^ 25.4%) (Fig. 2a). Although the efficacy was lower in patients with advanced liver disease, the therapy was superior to placebo in non-cirrhotic (28% (95%CI 24–33; *I*^2^ 91%) versus 20% (95%CI 17–23; *I*^2^ 61.7%)) and cirrhotic patients (16% (95%CI 11–21; *I*^2^ 59.3%) versus 12% (95%CI 8–17; *I*^2^ 23%)), respectively (Supplementary Fig. 7a,b). In addition to non-cirrhotic patients, the experimental therapy showed higher efficacy in trials with a duration  > 48 versus  ≤ 48 weeks, in studies showing  < 60% of diabetic population, and when based on antimetabolic drugs and FXR agonists (s).

**Fig. 3:**
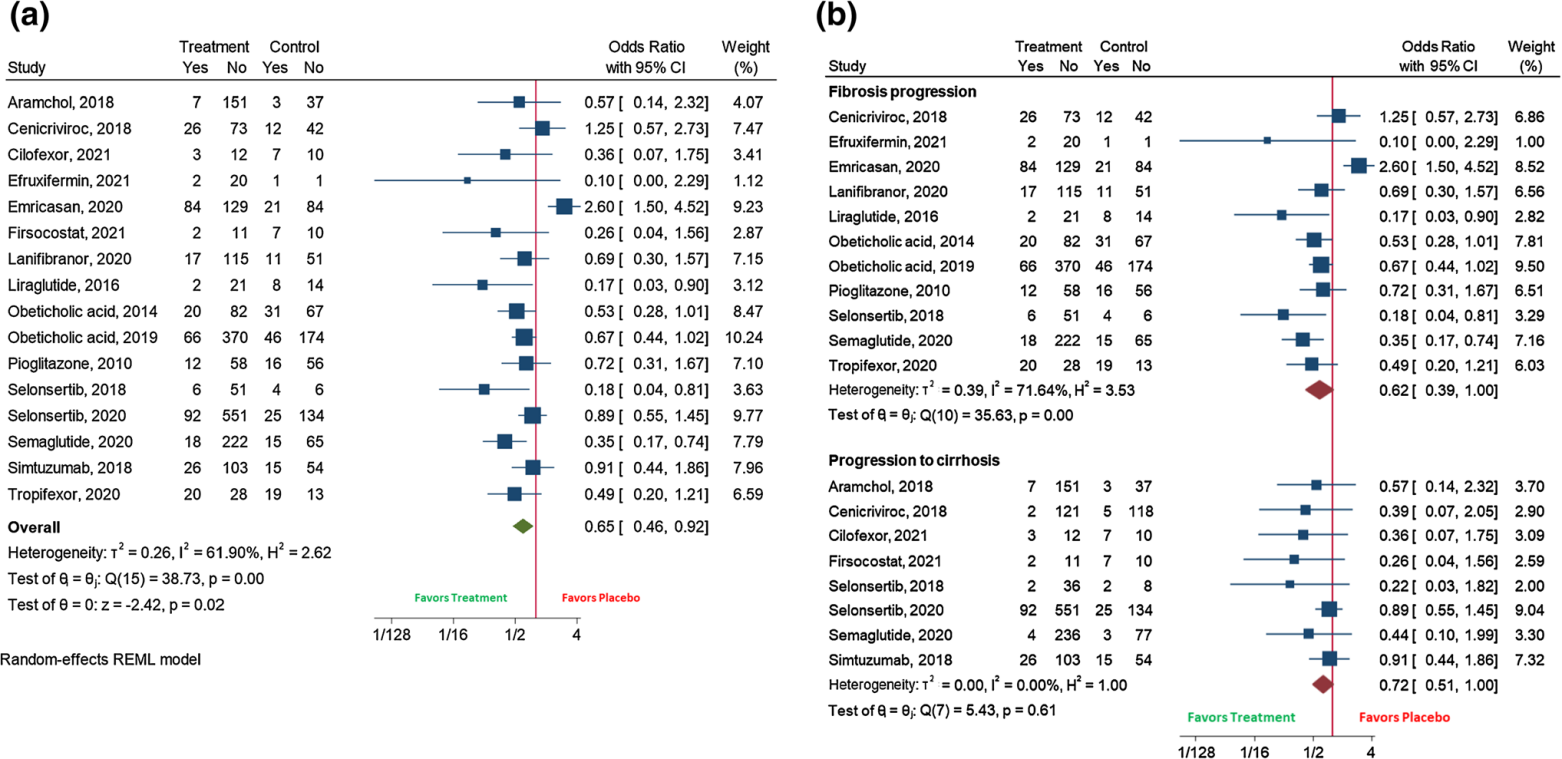
The effect of the experimental drug on: **A** Overall fibrosis progression; **B** Fibrosis progression *versus* progression to cirrhosis

Sixteen clinical trials (*N* = 3459 patients) assessed the fibrosis progression showing that patients receiving an experimental drug were more protected against this outcome (17% (95%CI 13–22); *I*^2^ 89.1%) than individuals under placebo (24% (95%CI 19–29); *I*^2^ 69.7%) (Supplementary Fig. 8a,b) (OR 0.65 (95%CI 0.46–0.92); *I*^2^ 61.9%) (Fig. 3a). Finally, when separating between fibrosis progression and progression towards cirrhosis, a similar protective role of the therapy was found [(OR 0.62 (95%CI 0.39–1.00); *I*^2^ 71.6%) and (OR 0.72 (95%CI 0.51–1.00); *I*^2^ 0%), respectively] (Fig. 3b).

### Other secondary endpoints

**Fig. 4:**
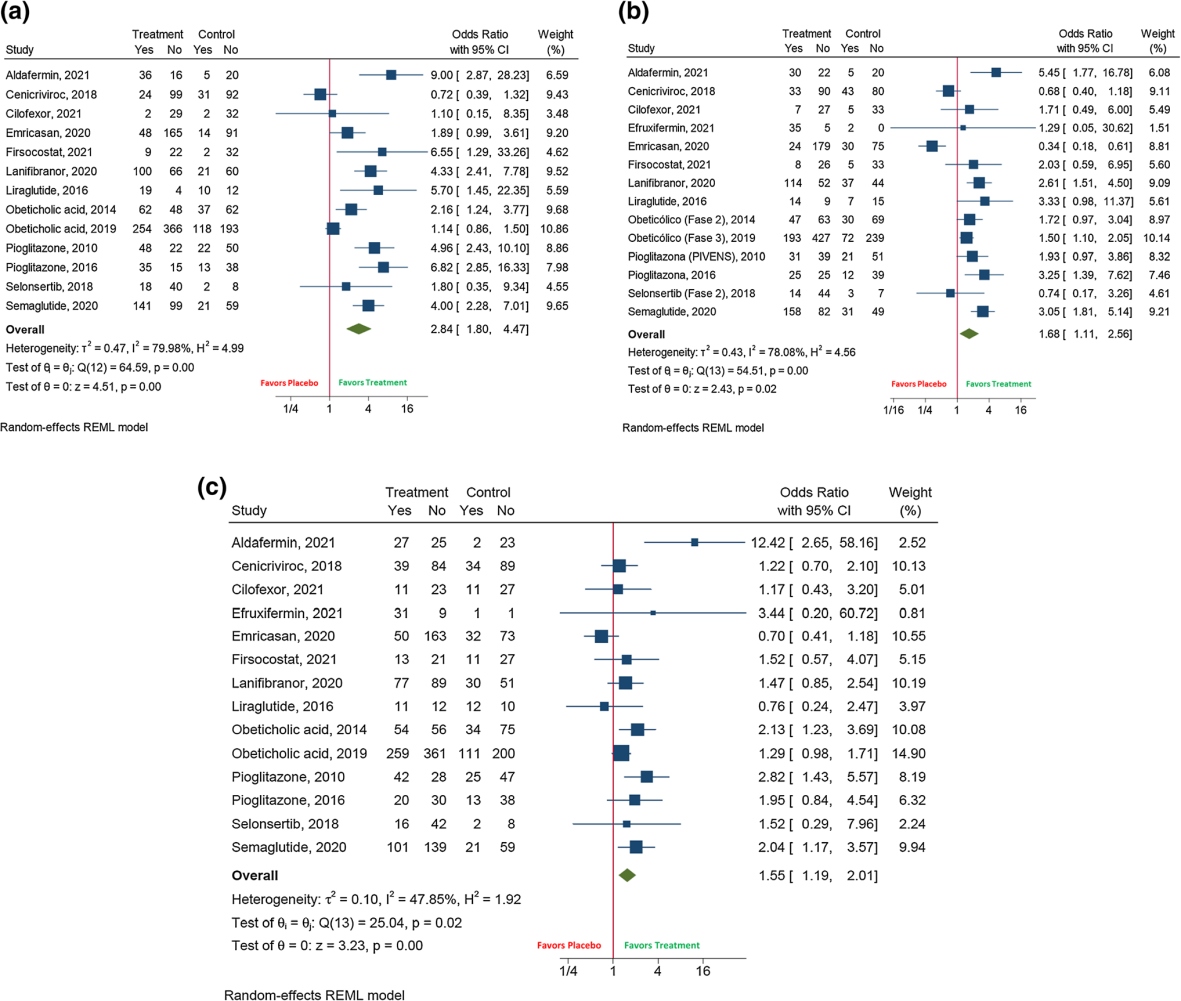
The effect of the experimental drug on: **A** Steatosis; **B** Ballooning; **C** Lobular inflammation

We also assessed the improvement of individual components of NAS (14 studies, 2876 patients). Regarding steatosis, the experimental treatment was associated with a higher rate of improvement (47% (95%CI 36–58); *I*^2^ 96%) than placebo (24% (95%CI 17–31); *I*^2^ 86.7%) (OR 2.84 (95%CI 1.80–4.47); *I*^2^ 80%) (Fig. 4a). Also, ballooning decreased more frequently in patients receiving the experimental treatment (40% (95%CI 29–52); *I*^2^ 96.5%) versus placebo (28% (95%CI 23–33); *I*^2^ 67.6%) (OR 1.68 (95%CI 1.11–2.56); *I*^2^ 78.1%) (Fig. 4b). Besides, the pooled efficacy of achieving lobular inflammation improvement was higher in patients receiving the drug (41% (95%CI 35–46); *I*^2^ 81.3%) than placebo (30% (95%CI 25–34); *I*^2^ 61%) (OR 1.55 (95%CI 1.19–2.01); *I*^2^ 47.9%) (Fig. 4c). On the other hand, steatosis (OR 0.34 (95%CI 0.22–0.52); *I*^2^ 0%) had a lower likelihood to progress in patients receiving any experimental therapy than placebo, although this did not occur with ballooning (OR 0.87 (95%CI 0.45–1.67); *I*^2^ 69%) and lobular inflammation (OR 0.71 (95%CI 0.34–1.46); *I*^2^ 79.8%). Otherwise, the occurrence of cirrhosis complications was not prevented when using an experimental treatment (*N* = 3) (OR 1.41 (95%CI 0.86–2.32); *I*^2^ 29.4%).

**Fig. 5:**
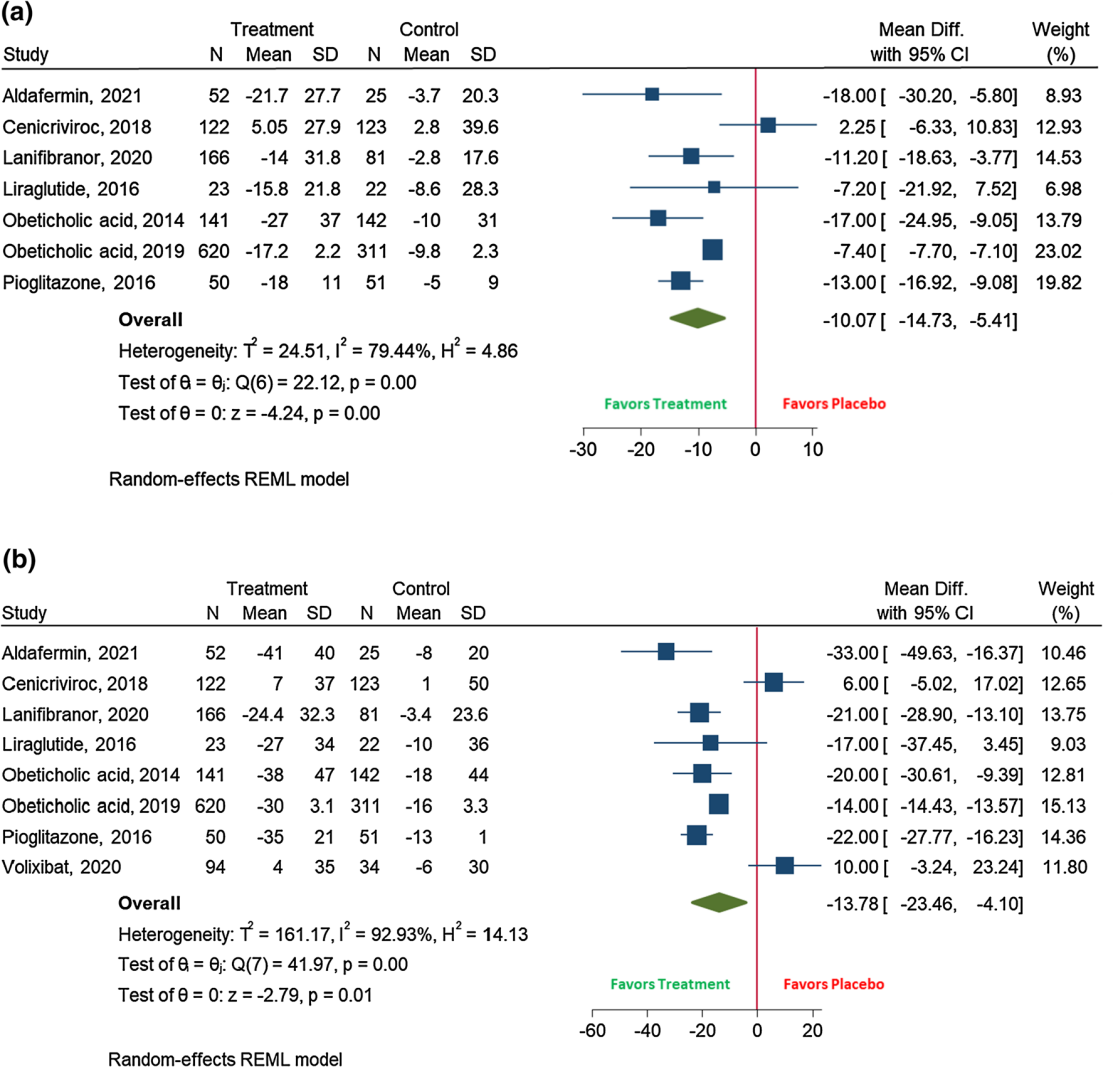
The effect of the experimental drug on: **A** AST levels; **B** ALT levels

Finally, the necro-inflammatory activity also improved when taking an experimental therapy. AST levels were significantly decreased in these individuals compared to those receiving placebo (mean difference –10.1 IU/L (95%CI (–14.7 to –5.4); *I*^2^ 79.4%)) (Fig. 5a). Similarly, ALT levels were found to be diminished under treatment (mean difference –13.8 IU/L (95%CI (–23.5 to –4.1); *I*^2^ 92.3%)) (Fig. 5b).

### Heterogeneity assessment and publication bias

The leave-one-out sensitivity analysis did not identify any single study that significantly contributed to the between-studies variability for NASH resolution (Supplementary Table 3), fibrosis improvement (Supplementary Table 4), and fibrosis progression (Supplementary Table 5). On the other hand, meta-regression showed no evidence of a differential effect of study-level characteristics on the impact of the outcomes, apart from baseline NAS for NASH resolution (*P* = 0.010) (Supplementary Table 6).

Publication bias was conducted by Egger’s test and funnel plot asymmetry. There was no formal evidence of publication bias for NASH resolution (*P* = 0.453) (Supplementary Fig. 9), improvement of NAS by  ≥ 2 points (*P* = 0.101) (Supplementary Fig. 10), fibrosis improvement  ≥ 1 stage (*P* = 0.451) (Supplementary Fig. 11), fibrosis progression (*P* = 0.105) (Supplementary Fig. 12), steatosis improvement (*P* = 0.312), ballooning improvement (*P* = 0.496), lobular inflammation improvement (*P* = 0.232), and biochemical response (*P* = 0.812 and *P* = 0.957 for AST and ALT, respectively).

## Discussion

Over the past five years, many clinical trials testing new drugs for NAFLD have been published [13–37. The scientific community has initially witnessed these preliminary data with enthusiasm and the final publication with a relative skepticism because of the suboptimal results, particularly for liver fibrosis improvement as an endpoint [38]. Our meta-analysis observed that taking an experimental therapy versus placebo increased the likelihood of resolute NASH and regress liver fibrosis. Despite the fact that only 5 of 76 studies demonstrated a beneficial effect of the therapy on NASH, the likelihood of NASH resolution was 60% higher than receiving placebo. In liver fibrosis, the likelihood of improving at least one stage was 30% higher with the therapy, although only 3 out of the 77 studies showed an individual benefit. Also, the individual components of NAS (steatosis, ballooning, and lobular inflammation), as well as the necro-inflammatory activity (evaluated by AST and ALT levels) significantly improved with the therapy. Of note, the percentage of NASH resolution and fibrosis regression for placebo was similar to that published in the literature [39], although recently it has been suggested a lower fibrosis progression rate in this group probably related to the number of patients without fibrosis [40]. Despite the global positive results, we found that the percentage of NASH resolution and fibrosis improvement was 19–28%, respectively, for experimental therapies based on biological plausibility, which are far from desirable. Due to its multifaceted nature [41], this fact mirrors the complexity of the underlying mechanisms of the pathogenesis of NAFLD.

We found that some baseline variables and features related to the clinical trial design influenced the likelihood of achieving the outcomes. First, FXR agonists and anti-metabolic drugs (including anti-diabetic therapies and PPAR agonists), and DNL-targeting therapies for NASH resolution, showed the highest efficacy for inflammation and fibrosis improvement. These findings have been documented in some studies [42, 43], although they showed limitations such as not assessing the clinical benefit in delaying disease progression and the baseline features impacting the efficacy of the drugs. Second, NASH resolution was easier to achieve in trials with non-cirrhotic patients, with baseline NAS < 5, with a low proportion of diabetic patients, and with a longer length of the therapy. These data did agree on variables associated with NASH-resolution after life-style intervention supporting a group of features defining more difficult-to-solve patients [44]. These characteristics were also more frequently associated with fibrosis improvement, with the exception of NAS. These results should make us to meditate on the design of NAFLD clinical trials and the adequacy of the endpoints to balance them with the prognostic relevance. On the one hand, the experimental therapy appeared to require at least 1 year to be effective. Thus, a longer duration than 48 weeks is preferred in NAFLD clinical trials. On the other hand, the drug effect was superior to placebo when achieving NASH resolution and fibrosis improvement, but it was not in trials including more severe patients. Therefore, NASH resolution should be required for the experimental therapy in non-cirrhotic patients and in those with a baseline NAS < 5, but questionable for individuals showing a NAS ≥ 5 and, especially, for cirrhotic patients since most of them have lost some of the single components of NAS [45]. Similarly, although desirable, fibrosis improvement is not a realistic aim for cirrhotic patients using the current experimental therapies, according to our results. Instead, clinical trials on cirrhotic patients should focus on preventing portal hypertension, hepatocellular carcinoma occurrence, and mortality, extending the treatment course, rather than in the regression of liver disease. Therefore, we should make efforts to redirect the design and selection criteria of clinical trials because some potentially useful drugs could be discarded too early.

NAFLD clinical trials should report a minimum of information about all relevant aspects that could impact on the efficacy of the experimental drug tested [7]. In this setting, results about efficacy tend to focus on the histological improvement (e.g., NASH or fibrosis), but frequently fail to mention data associated with the prevention of its progression. For example, in our meta-analysis, only 4 and 16 of 27 clinical trials reported information about worsening of NASH and fibrosis, respectively. Considering this, our results indicate that patients receiving therapy were protected against NASH worsening and/or fibrosis progression. In other liver diseases, the treatment aims mainly to eliminate (e.g., hepatitis C) or control the etiology (e.g., hepatitis B, autoimmune hepatitis) but does not reverse liver fibrosis, which is a consequence that requires an extended follow-up [46–48]. Instead, NAFLD clinical trials require an early resolution of NASH or fibrosis improvement to be considered a success. Given the nescience regarding in whom fibrosis regression can be expected and how quickly it occurs, we should consider halting the disease as a relevant outcome and, thus, complementary to the improvement of liver disease. Therefore, we encourage NAFLD clinical trials to report essential information about the progression of the disease to have an overall vision of the efficacy of experimental drugs.

Beyond the strengths, our meta-analysis also has some limitations. First, the interpretation of some results could be challenging because of the different mechanisms of action of included drugs. However, this kind of approach has been done for other therapeutic areas (e.g., biologics in ulcerative colitis [49]) and could be interesting to provide additional data to guide the NAFLD drug pipeline properly. Second, studies reporting cirrhotic-related outcomes were scarce, precluding making robust analysis. Third, some baseline variables, such as T2DM or NAS, were categorized. This usual aspect allows making subgroup analyses or a meta-regression in the absence of the individual data but with a limitation in the interpretation.

In conclusion, developing therapeutic strategies to revert or, at least, slow down steatohepatitis and fibrosis progression as much as possible in NAFLD is an unmet need. This meta-analysis provides information about the efficacy of the therapy versus placebo by comparing different and combined trial outcomes such as NASH resolution, fibrosis improvement, and NAS and fibrosis worsening. Given that novel pharmacological agents focused on NASH resolution and liver fibrosis regression are expected to be available in the upcoming years, changes in the experimental design and selection criteria of the clinical trials may increase the ability to demonstrate efficacy.

## Supplementary Information

Below is the link to the electronic supplementary material.

Supplementary file1 (PPTX 40 KB)

Supplementary file2 (TIF 3134 KB)

Supplementary file3 (TIF 3134 KB)

Supplementary file4 (TIF 3134 KB)

Supplementary file5 (PPTX 60 KB)

Supplementary file6 (PPTX 60 KB)

Supplementary file7 (PPTX 63 KB)

Supplementary file8 (PPTX 55 KB)

Supplementary file9 (PPTX 55 KB)

Supplementary file10 (PPTX 44 KB)

Supplementary file11 (PPTX 44 KB)

Supplementary file12 (PPTX 41 KB)

Supplementary file13 (PPTX 61 KB)

Supplementary file14 (PPTX 61 KB)

Supplementary file15 (PPTX 56 KB)

Supplementary file16 (PPTX 57 KB)

Supplementary file17 (TIF 3134 KB)

Supplementary file18 (DOCX 13 KB)

Supplementary file19 (DOCX 20 KB)

Supplementary file20 (DOCX 14 KB)

Supplementary file21 (DOCX 14 KB)

Supplementary file22 (DOCX 14 KB)

Supplementary file23 (DOCX 13 KB)

## Abbreviations

BMI: Body mass index
CI: Confidence interval
DNL: De novo lipogenesis
NAFL: Non-alcoholic fatty liver
NAFLD: Non-alcoholic fatty liver disease
NASH: Non-alcoholic steatohepatitis
NAS: NAFLD activity score
OR: Odds ratio
T2DM: Type 2 diabetes mellitus
