# Supplementary material for: Systematic review and meta-analysis: analysis of variables influencing the interpretation of clinical trial results in NAFLD
Source: J Gastroenterol. 2022 Mar 24;57(5):357–71. doi: 10.1007/s00535-022-01860-0 (PMC9016009; doi:10.1007/s00535-022-01860-0)
Supplement: Supplementary file 1 — Supplementary file1 (PPTX 40 KB) [file 535_2022_1860_MOESM1_ESM.pptx]

## Slide 1
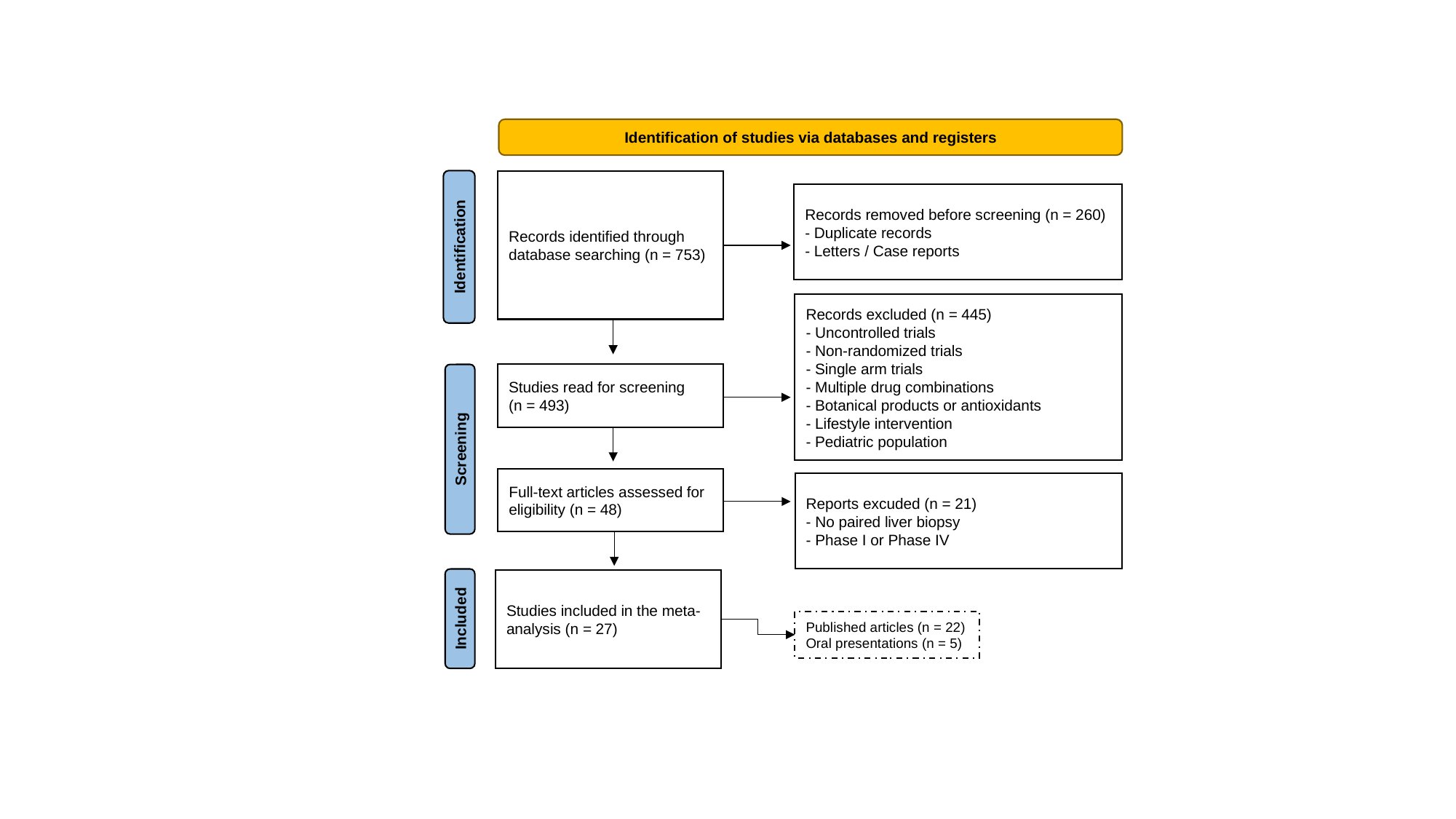

Identification of studies via databases and registers
Records identified through database searching (n = 753)
Records removed before screening (n = 260)
- Duplicate records
- Letters / Case reports
Identification
Records excluded (n = 445)
- Uncontrolled trials
- Non-randomized trials
- Single arm trials
- Multiple drug combinations
- Botanical products or antioxidants
- Lifestyle intervention
- Pediatric population
Studies read for screening
(n = 493)
Screening
Full-text articles assessed for eligibility (n = 48)
Reports excuded (n = 21)
- No paired liver biopsy
- Phase I or Phase IV
Studies included in the meta-analysis (n = 27)
Included
Published articles (n = 22)
Oral presentations (n = 5)
