# Supplementary figures and images for: Systematic review and meta-analysis: analysis of variables influencing the interpretation of clinical trial results in NAFLD
Source: J Gastroenterol. 2022 Mar 24;57(5):357–71. doi: 10.1007/s00535-022-01860-0 (PMC9016009; doi:10.1007/s00535-022-01860-0)

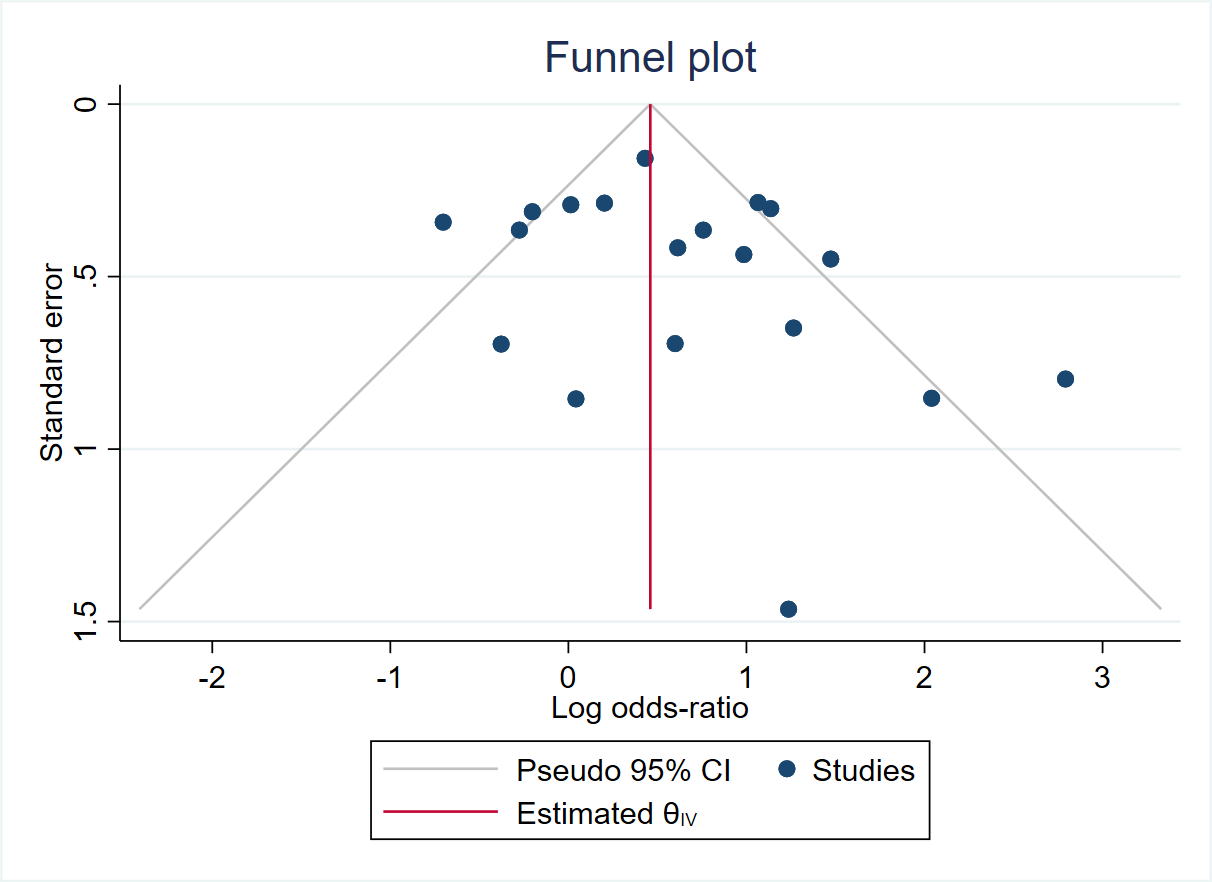

Supplement: Supplementary file 2 — Supplementary file2 (TIF 3134 KB) [file 535_2022_1860_MOESM2_ESM.tif]

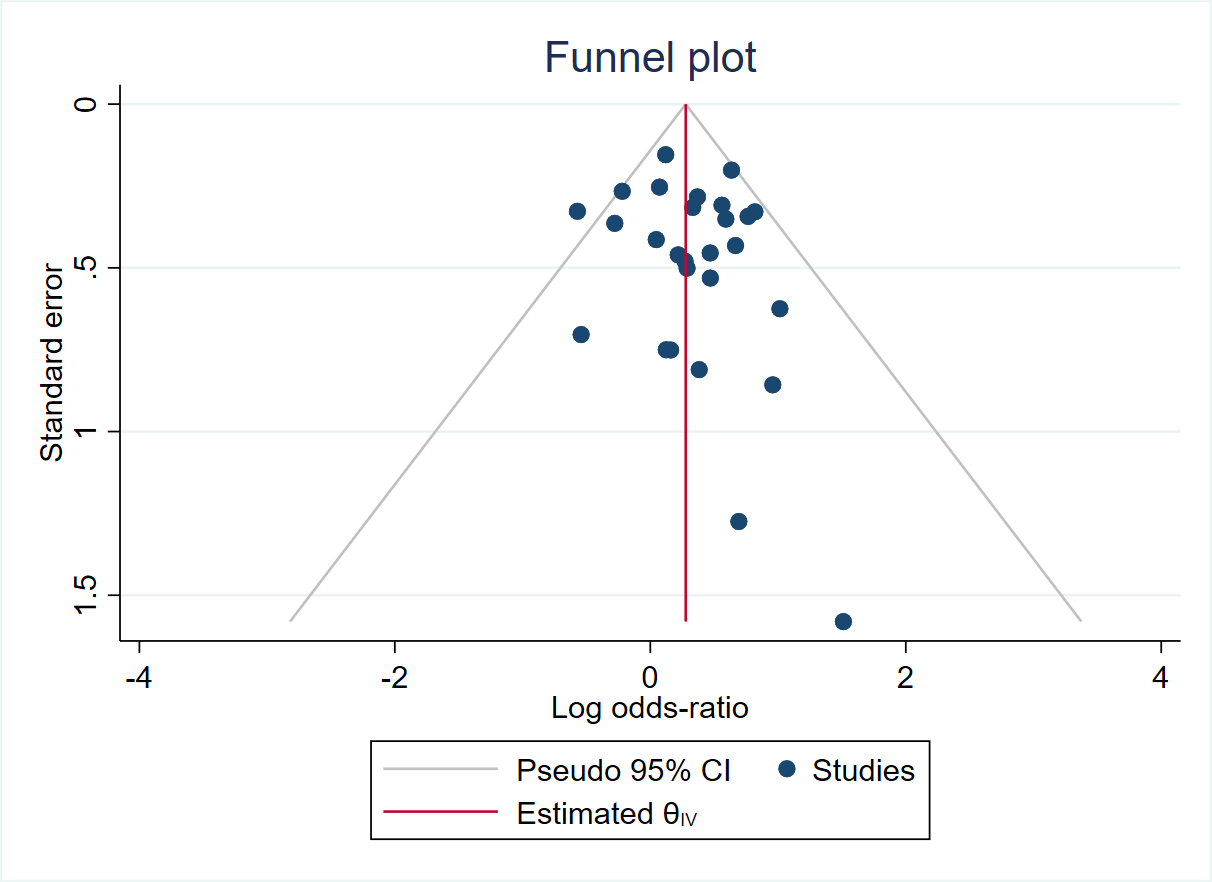

Supplement: Supplementary file 3 — Supplementary file3 (TIF 3134 KB) [file 535_2022_1860_MOESM3_ESM.tif]

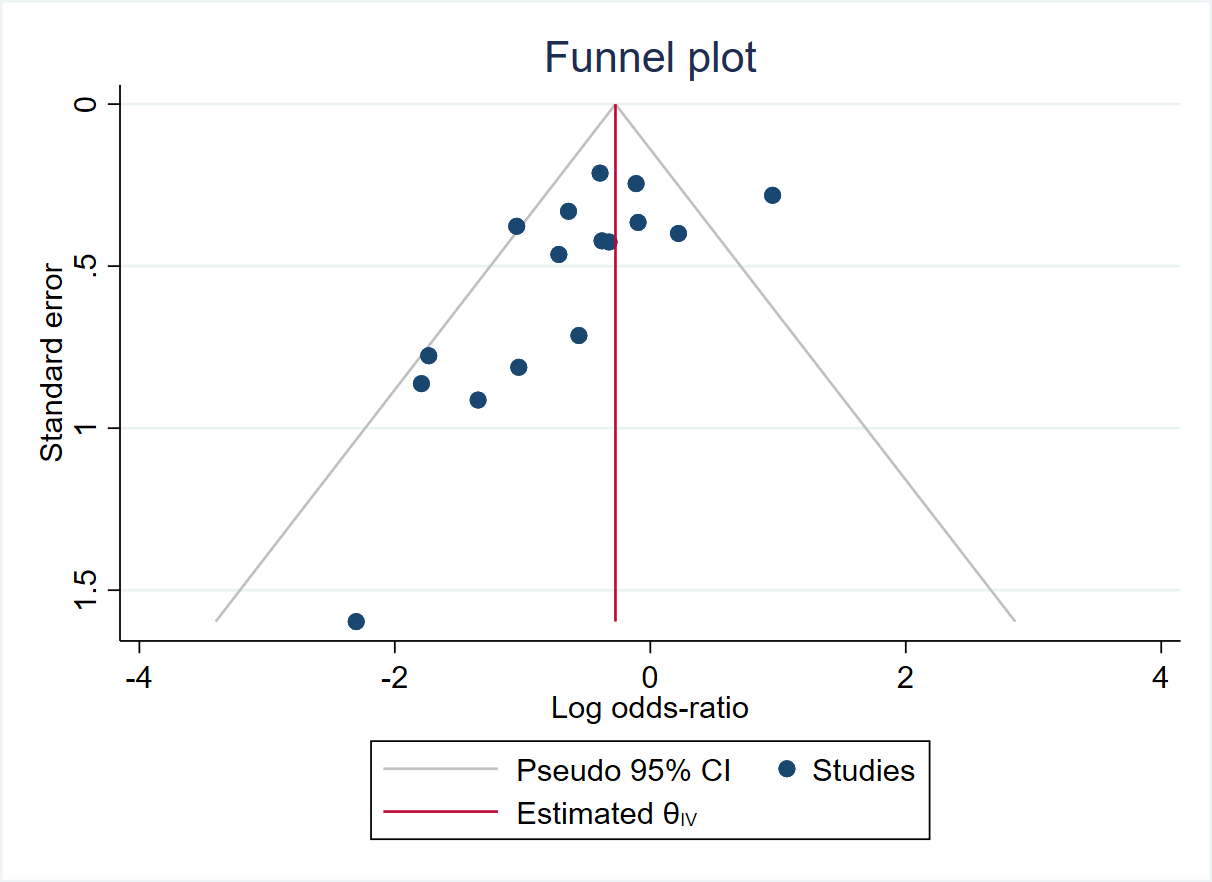

Supplement: Supplementary file 4 — Supplementary file4 (TIF 3134 KB) [file 535_2022_1860_MOESM4_ESM.tif]

## Slide 1
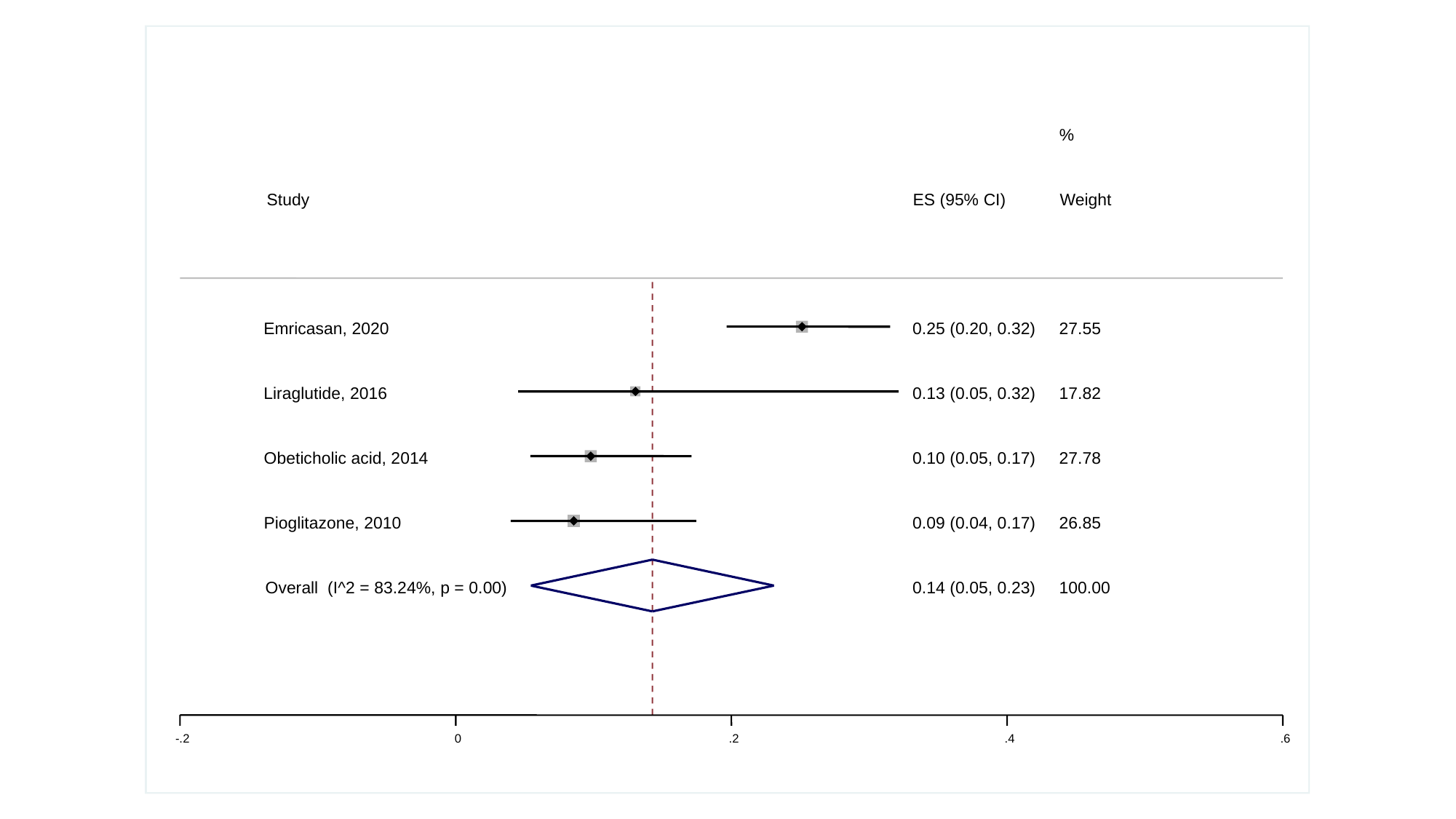

%
Study
ES (95% CI)
Weight
Emricasan, 2020
0.25 (0.20, 0.32)
27.55
Liraglutide, 2016
0.13 (0.05, 0.32)
17.82
Obeticholic acid, 2014
0.10 (0.05, 0.17)
27.78
Pioglitazone, 2010
0.09 (0.04, 0.17)
26.85
Overall (I^2 = 83.24%, p = 0.00)
0.14 (0.05, 0.23)
100.00
-.2
0
.2
.4
.6

Supplement: Supplementary file 10 — Supplementary file10 (PPTX 44 KB) [file 535_2022_1860_MOESM10_ESM.pptx]

## Slide 1
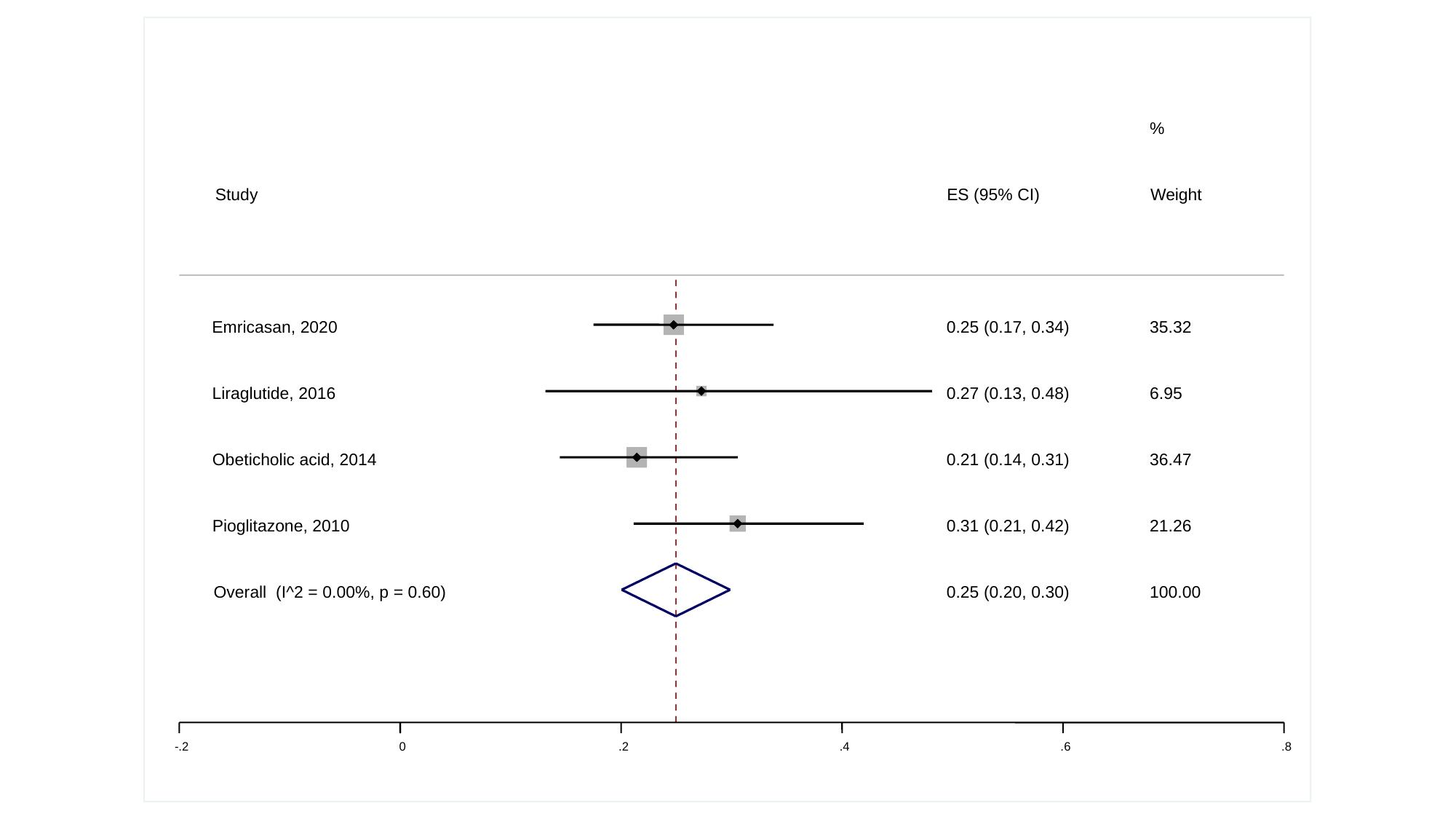

%
Study
ES (95% CI)
Weight
Emricasan, 2020
0.25 (0.17, 0.34)
35.32
Liraglutide, 2016
0.27 (0.13, 0.48)
6.95
Obeticholic acid, 2014
0.21 (0.14, 0.31)
36.47
Pioglitazone, 2010
0.31 (0.21, 0.42)
21.26
Overall (I^2 = 0.00%, p = 0.60)
0.25 (0.20, 0.30)
100.00
-.2
0
.2
.4
.6
.8

Supplement: Supplementary file 11 — Supplementary file11 (PPTX 44 KB) [file 535_2022_1860_MOESM11_ESM.pptx]

## Slide 1
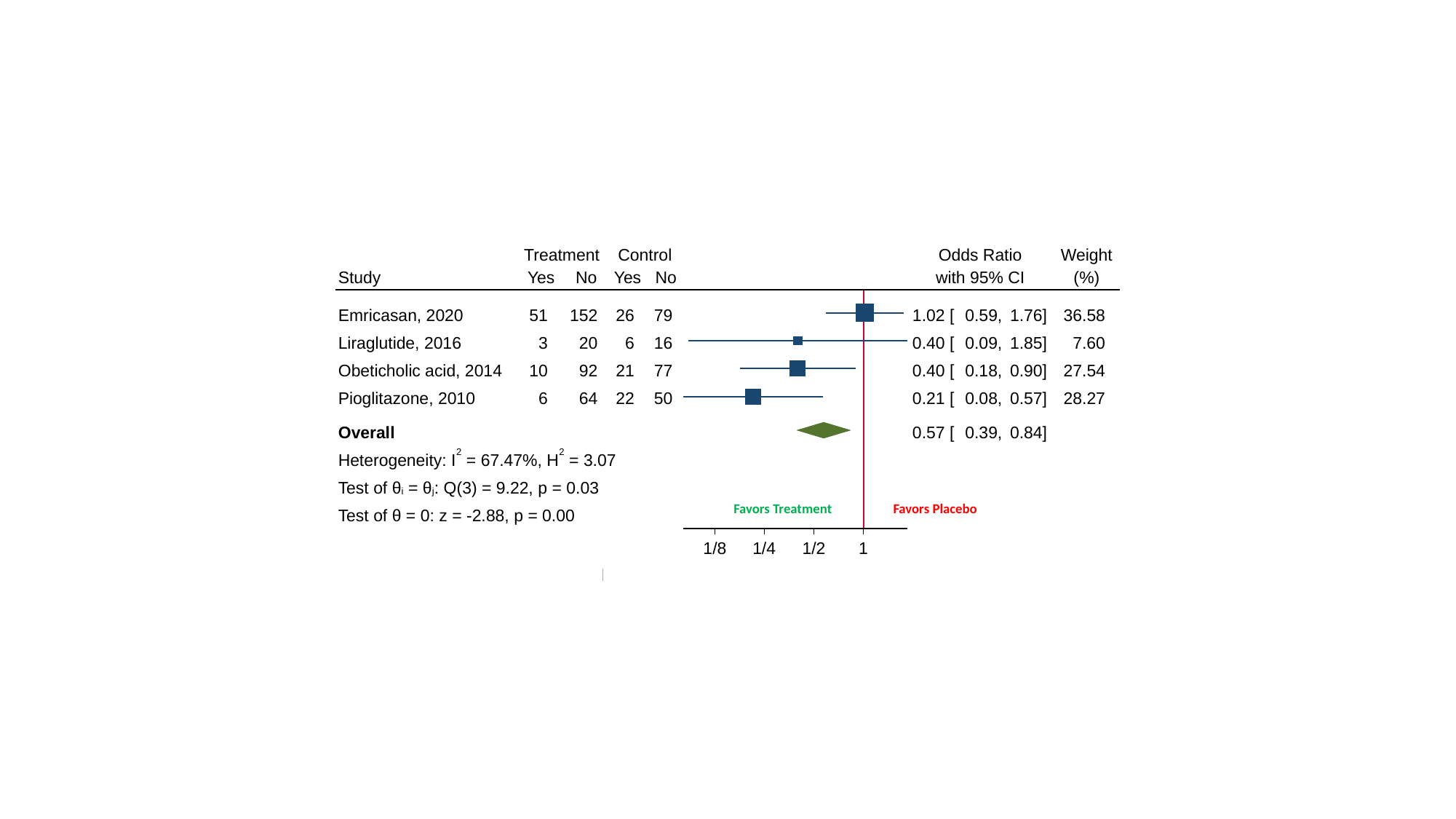

Favors Treatment
Favors Placebo

Supplement: Supplementary file 12 — Supplementary file12 (PPTX 41 KB) [file 535_2022_1860_MOESM12_ESM.pptx]

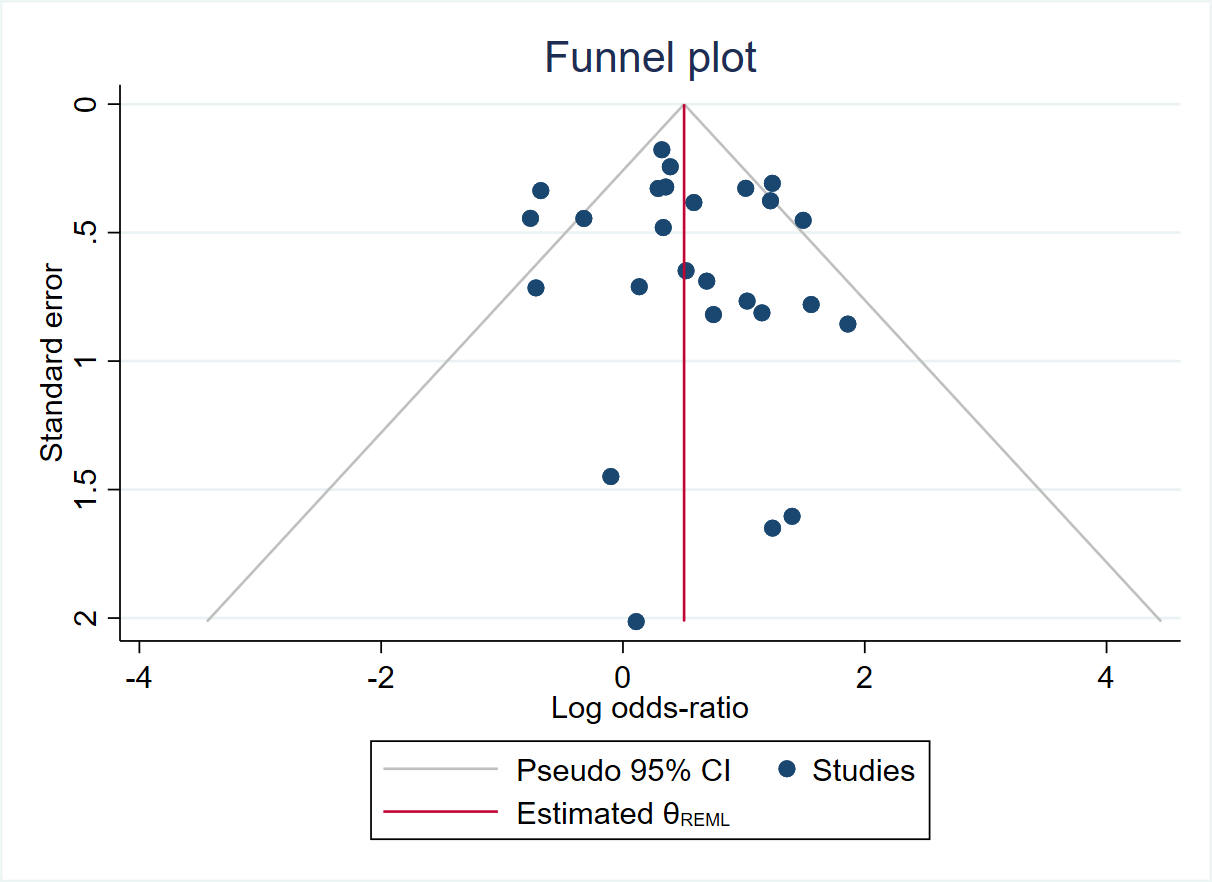

Supplement: Supplementary file 17 — Supplementary file17 (TIF 3134 KB) [file 535_2022_1860_MOESM17_ESM.tif]
