# Supplementary material for: Systematic review and meta-analysis: analysis of variables influencing the interpretation of clinical trial results in NAFLD
Source: J Gastroenterol. 2022 Mar 24;57(5):357–71. doi: 10.1007/s00535-022-01860-0 (PMC9016009; doi:10.1007/s00535-022-01860-0)
Supplement: Supplementary file 5 — Supplementary file5 (PPTX 60 KB) [file 535_2022_1860_MOESM5_ESM.pptx]

## Slide 1
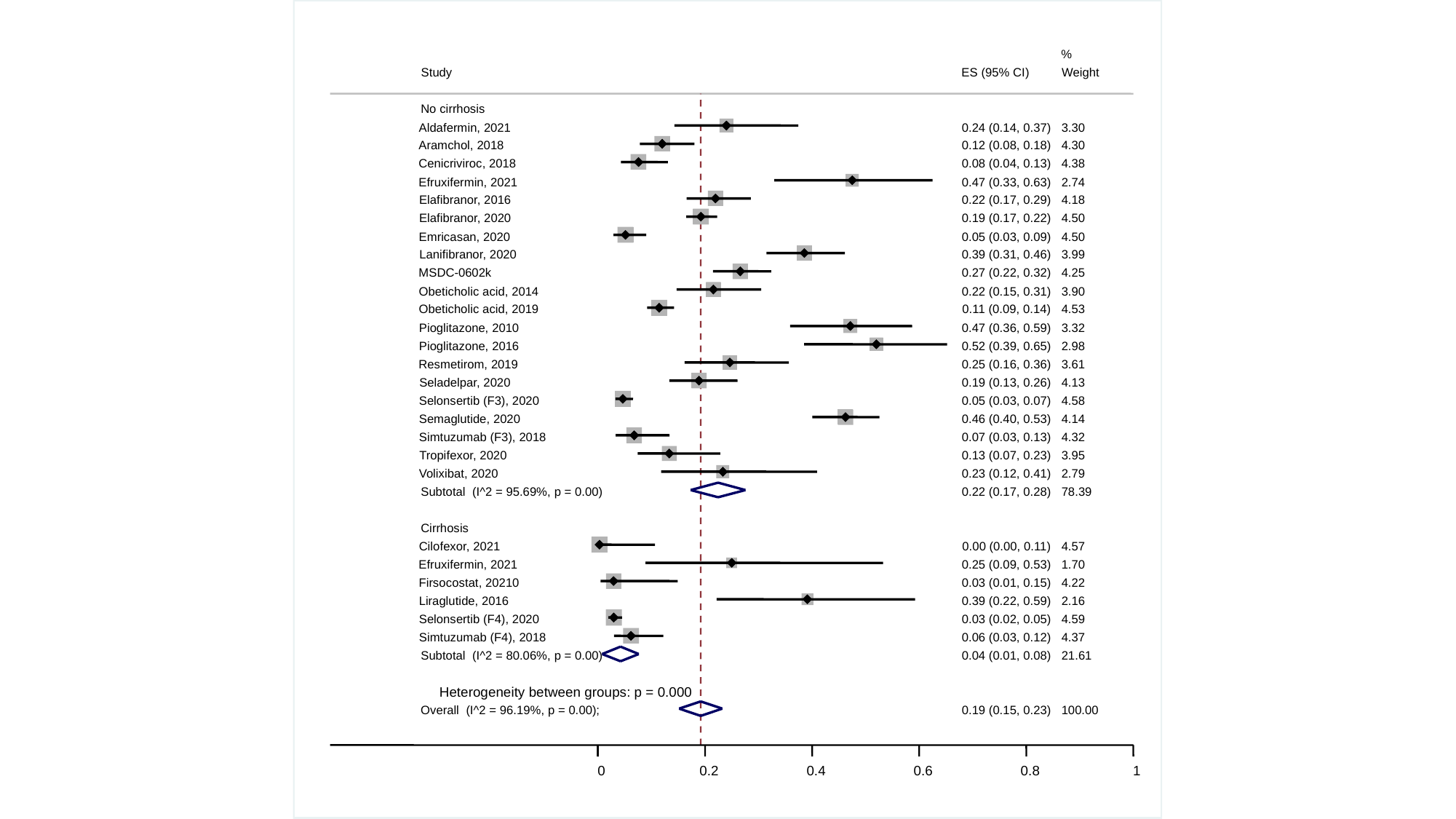

Study
ES (95% CI)
No cirrhosis
Aldafermin, 2021
Aramchol, 2018
0.12 (0.08, 0.18)
Cenicriviroc, 2018
0.08 (0.04, 0.13)
Efruxifermin, 2021
Elafibranor, 2016
0.22 (0.17, 0.29)
Elafibranor, 2020
Emricasan, 2020
Lanifibranor, 2020
MSDC-0602k
Obeticholic acid, 2014
0.22 (0.15, 0.31)
Obeticholic acid, 2019
0.11 (0.09, 0.14)
Pioglitazone, 2010
0.47 (0.36, 0.59)
Pioglitazone, 2016
Resmetirom, 2019
0.25 (0.16, 0.36)
Seladelpar, 2020
Selonsertib (F3), 2020
Semaglutide, 2020
0.46 (0.40, 0.53)
Simtuzumab (F3), 2018
0.07 (0.03, 0.13)
Tropifexor, 2020
0.13 (0.07, 0.23)
Volixibat, 2020
Subtotal (I^2 = 95.69%, p = 0.00)
0.22 (0.17, 0.28)
Cirrhosis
Cilofexor, 2021
0.00 (0.00, 0.11)
Efruxifermin, 2021
Firsocostat, 20210
0.03 (0.01, 0.15)
Liraglutide, 2016
0.39 (0.22, 0.59)
Selonsertib (F4), 2020
Simtuzumab (F4), 2018
Subtotal (I^2 = 80.06%, p = 0.00)
0.04 (0.01, 0.08)
Heterogeneity between groups: p = 0.000
Overall (I^2 = 96.19%, p = 0.00);
0.19 (0.15, 0.23)
%
Weight
0.24 (0.14, 0.37)
3.30
4.30
4.38
0.47 (0.33, 0.63)
2.74
4.18
0.19 (0.17, 0.22)
4.50
0.05 (0.03, 0.09)
4.50
0.39 (0.31, 0.46)
3.99
0.27 (0.22, 0.32)
4.25
3.90
4.53
3.32
0.52 (0.39, 0.65)
2.98
3.61
0.19 (0.13, 0.26)
4.13
0.05 (0.03, 0.07)
4.58
4.14
4.32
3.95
0.23 (0.12, 0.41)
2.79
78.39
4.57
0.25 (0.09, 0.53)
1.70
4.22
2.16
0.03 (0.02, 0.05)
4.59
0.06 (0.03, 0.12)
4.37
21.61
100.00
0
0.2
0.4
0.6
0.8
1
