# Supplementary material for: Systematic review and meta-analysis: analysis of variables influencing the interpretation of clinical trial results in NAFLD
Source: J Gastroenterol. 2022 Mar 24;57(5):357–71. doi: 10.1007/s00535-022-01860-0 (PMC9016009; doi:10.1007/s00535-022-01860-0)
Supplement: Supplementary file 6 — Supplementary file6 (PPTX 60 KB) [file 535_2022_1860_MOESM6_ESM.pptx]

## Slide 1
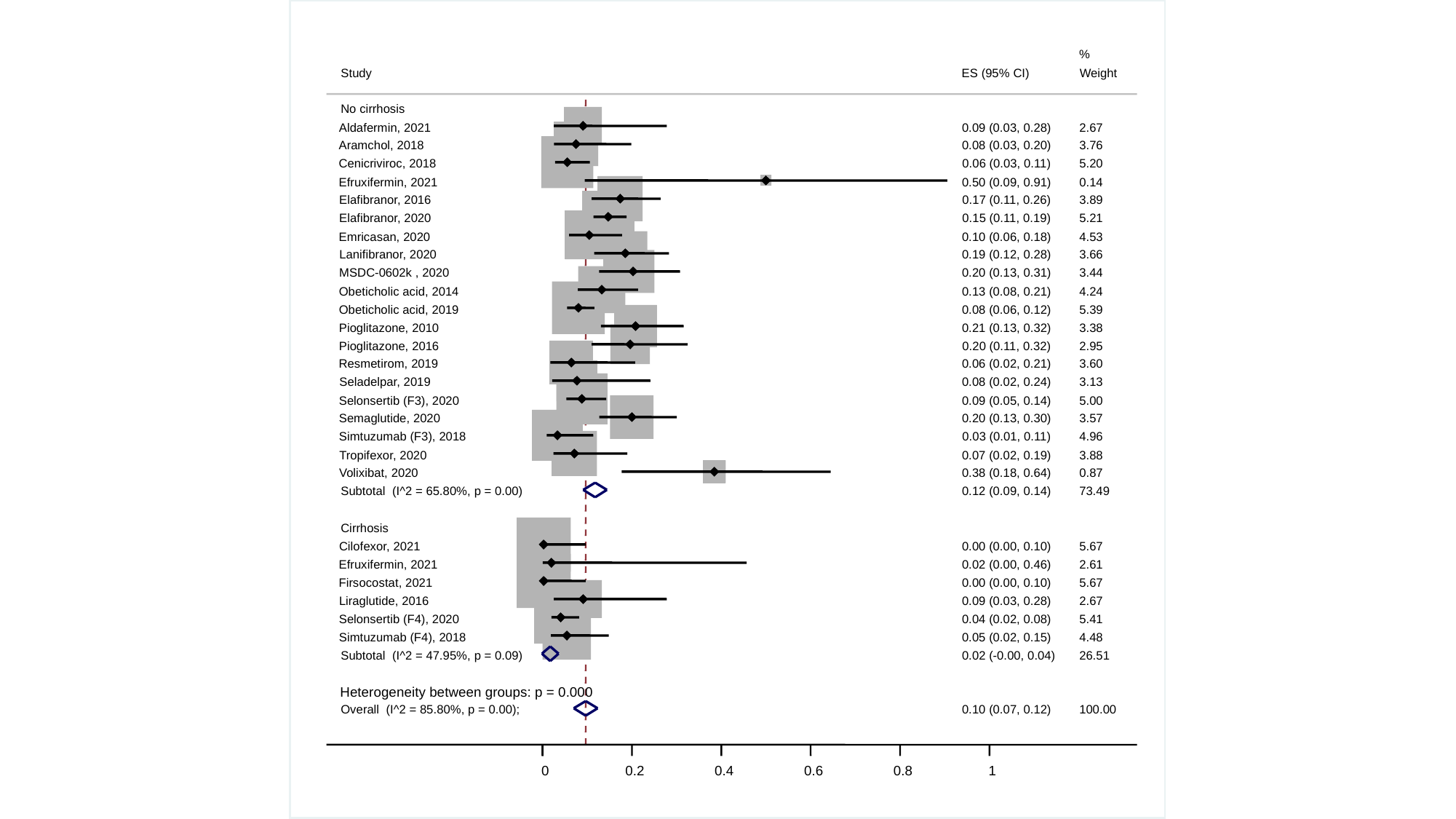

Study
ES (95% CI)
No cirrhosis
Aldafermin, 2021
Aramchol, 2018
Cenicriviroc, 2018
Efruxifermin, 2021
0.50 (0.09, 0.91)
Elafibranor, 2016
Elafibranor, 2020
Emricasan, 2020
0.10 (0.06, 0.18)
Lanifibranor, 2020
0.19 (0.12, 0.28)
MSDC-0602k , 2020
0.20 (0.13, 0.31)
Obeticholic acid, 2014
Obeticholic acid, 2019
Pioglitazone, 2010
Pioglitazone, 2016
0.20 (0.11, 0.32)
Resmetirom, 2019
Seladelpar, 2019
0.08 (0.02, 0.24)
Selonsertib (F3), 2020
0.09 (0.05, 0.14)
Semaglutide, 2020
0.20 (0.13, 0.30)
Simtuzumab (F3), 2018
0.03 (0.01, 0.11)
Tropifexor, 2020
Volixibat, 2020
0.38 (0.18, 0.64)
Subtotal (I^2 = 65.80%, p = 0.00)
0.12 (0.09, 0.14)
Cirrhosis
Cilofexor, 2021
0.00 (0.00, 0.10)
Efruxifermin, 2021
Firsocostat, 2021
0.00 (0.00, 0.10)
Liraglutide, 2016
0.09 (0.03, 0.28)
Selonsertib (F4), 2020
0.04 (0.02, 0.08)
Simtuzumab (F4), 2018
0.05 (0.02, 0.15)
Subtotal (I^2 = 47.95%, p = 0.09)
Heterogeneity between groups: p = 0.000
Overall (I^2 = 85.80%, p = 0.00);
0.10 (0.07, 0.12)
%
Weight
0.09 (0.03, 0.28)
2.67
0.08 (0.03, 0.20)
3.76
0.06 (0.03, 0.11)
5.20
0.14
0.17 (0.11, 0.26)
3.89
0.15 (0.11, 0.19)
5.21
4.53
3.66
3.44
0.13 (0.08, 0.21)
4.24
0.08 (0.06, 0.12)
5.39
0.21 (0.13, 0.32)
3.38
2.95
0.06 (0.02, 0.21)
3.60
3.13
5.00
3.57
4.96
0.07 (0.02, 0.19)
3.88
0.87
73.49
5.67
0.02 (0.00, 0.46)
2.61
5.67
2.67
5.41
4.48
0.02 (-0.00, 0.04)
26.51
100.00
0
0.2
0.4
0.6
0.8
1
