# Supplementary material for: Systematic review and meta-analysis: analysis of variables influencing the interpretation of clinical trial results in NAFLD
Source: J Gastroenterol. 2022 Mar 24;57(5):357–71. doi: 10.1007/s00535-022-01860-0 (PMC9016009; doi:10.1007/s00535-022-01860-0)
Supplement: Supplementary file 7 — Supplementary file7 (PPTX 63 KB) [file 535_2022_1860_MOESM7_ESM.pptx]

## Slide 1
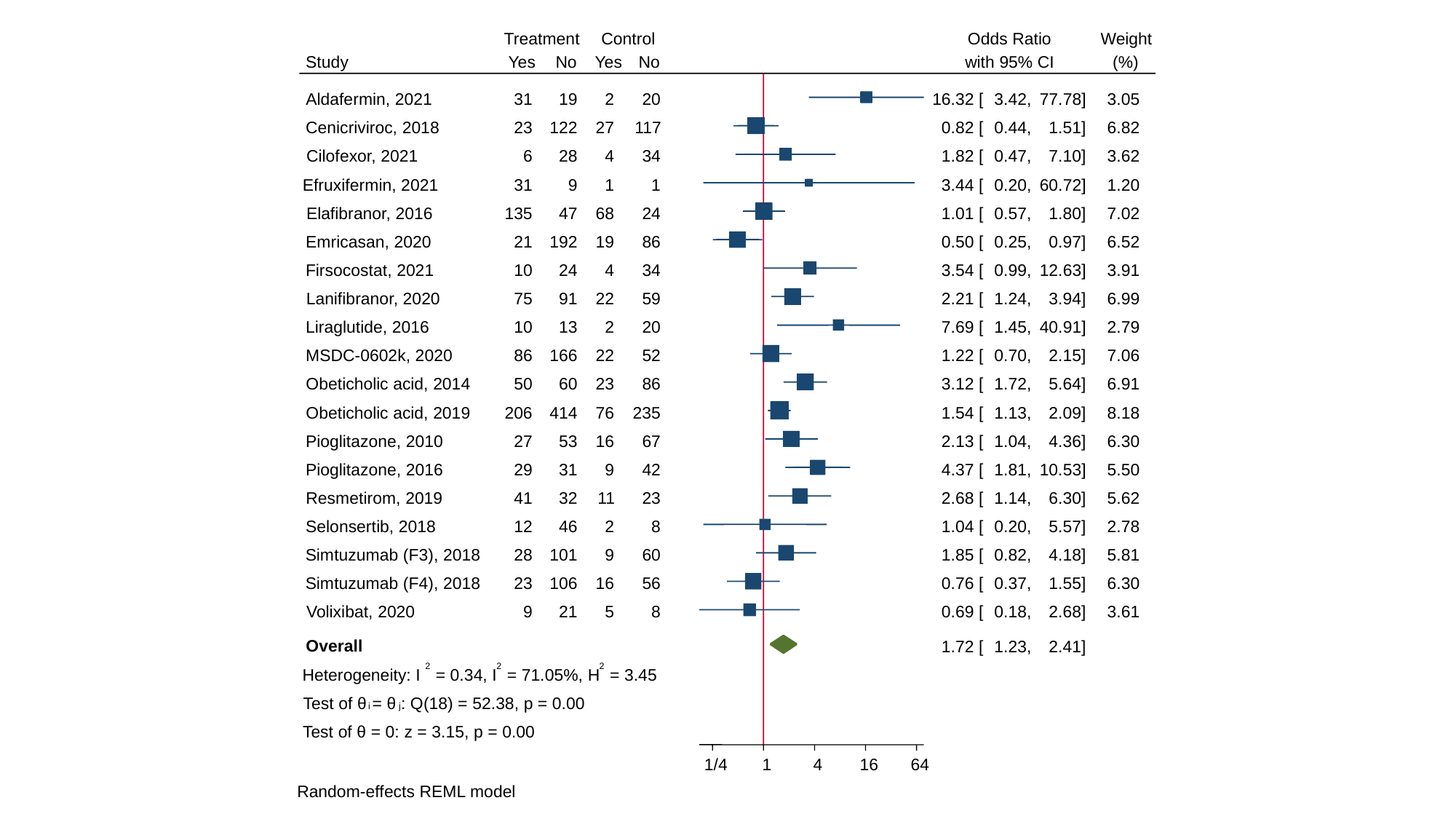

Treatment
Control
Study
Yes
No
Yes
No
Aldafermin, 2021
31
19
2
20
Cenicriviroc, 2018
23
122
27
117
Cilofexor, 2021
6
28
4
34
Efruxifermin, 2021
31
9
1
1
Elafibranor, 2016
135
47
68
24
Emricasan, 2020
21
192
19
86
Firsocostat, 2021
10
24
4
34
Lanifibranor, 2020
75
91
22
59
Liraglutide, 2016
10
13
2
20
MSDC-0602k, 2020
86
166
22
52
Obeticholic acid, 2014
50
60
23
86
Obeticholic acid, 2019
206
414
76
235
Pioglitazone, 2010
27
53
16
67
Pioglitazone, 2016
29
31
9
42
Resmetirom, 2019
41
32
11
23
Selonsertib, 2018
12
46
2
8
Simtuzumab (F3), 2018
28
101
9
60
Simtuzumab (F4), 2018
23
106
16
56
Volixibat, 2020
9
21
5
8
Overall
2
2
2
Heterogeneity: I
 = 0.34, I
 = 71.05%, H
 = 3.45
Test of θ
 = θ
: Q(18) = 52.38, p = 0.00
i
j
Test of θ = 0: z = 3.15, p = 0.00
Odds Ratio
Weight
with 95% CI
(%)
16.32 [
3.42,
77.78]
3.05
0.82 [
0.44,
1.51]
6.82
1.82 [
0.47,
7.10]
3.62
3.44 [
0.20,
60.72]
1.20
1.01 [
0.57,
1.80]
7.02
0.50 [
0.25,
0.97]
6.52
3.54 [
0.99,
12.63]
3.91
2.21 [
1.24,
3.94]
6.99
7.69 [
1.45,
40.91]
2.79
1.22 [
0.70,
2.15]
7.06
3.12 [
1.72,
5.64]
6.91
1.54 [
1.13,
2.09]
8.18
2.13 [
1.04,
4.36]
6.30
4.37 [
1.81,
10.53]
5.50
2.68 [
1.14,
6.30]
5.62
1.04 [
0.20,
5.57]
2.78
1.85 [
0.82,
4.18]
5.81
0.76 [
0.37,
1.55]
6.30
0.69 [
0.18,
2.68]
3.61
1.72 [
1.23,
2.41]
1/4
1
4
16
64
Random-effects REML model
Favors Treatment
Favors Placebo
