# Supplementary material for: Systematic review and meta-analysis: analysis of variables influencing the interpretation of clinical trial results in NAFLD
Source: J Gastroenterol. 2022 Mar 24;57(5):357–71. doi: 10.1007/s00535-022-01860-0 (PMC9016009; doi:10.1007/s00535-022-01860-0)
Supplement: Supplementary file 8 — Supplementary file8 (PPTX 55 KB) [file 535_2022_1860_MOESM8_ESM.pptx]

## Slide 1
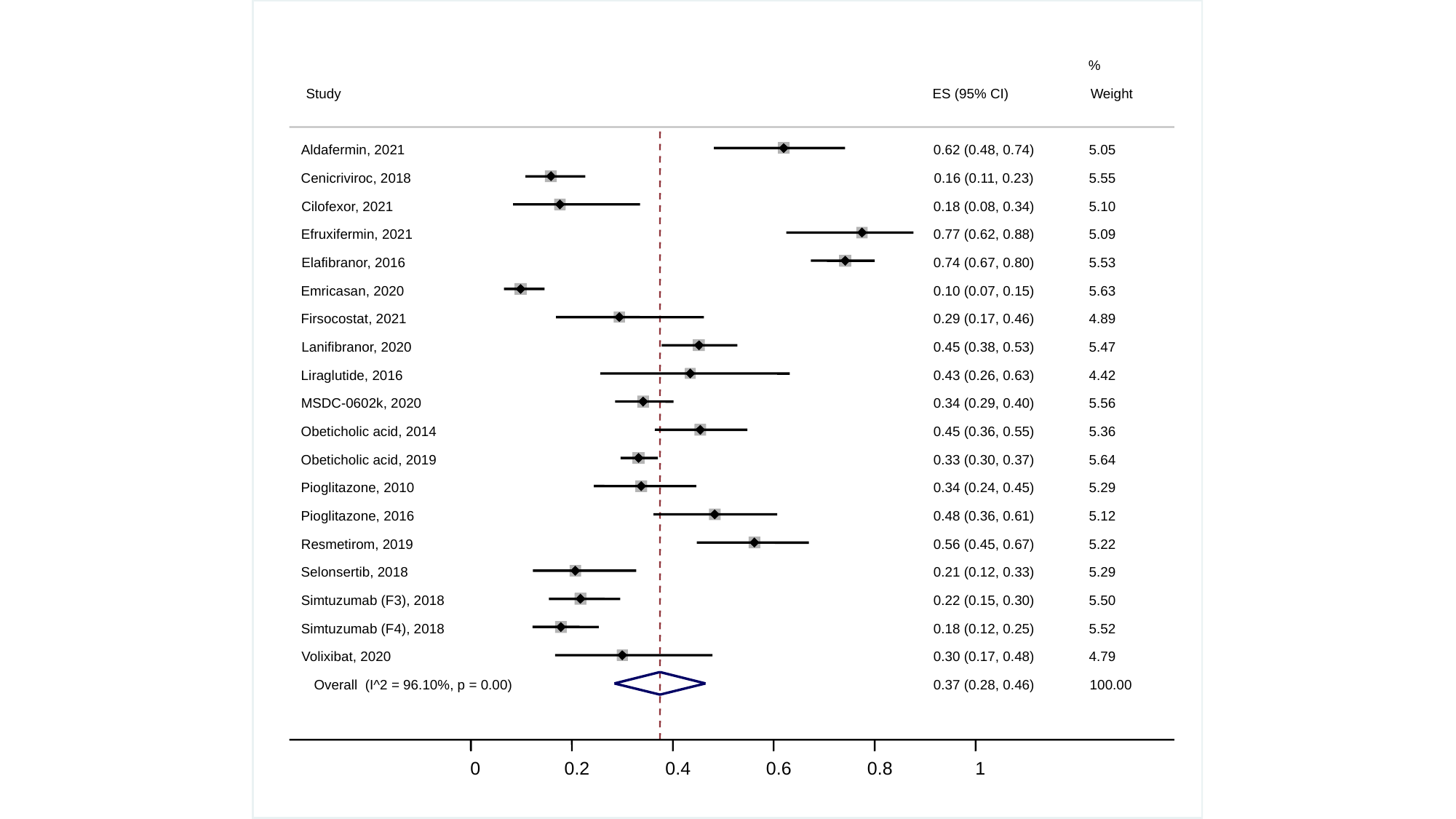

%
Study
ES (95% CI)
Weight
Aldafermin, 2021
0.62 (0.48, 0.74)
5.05
Cenicriviroc, 2018
0.16 (0.11, 0.23)
5.55
Cilofexor, 2021
0.18 (0.08, 0.34)
5.10
Efruxifermin, 2021
0.77 (0.62, 0.88)
5.09
Elafibranor, 2016
0.74 (0.67, 0.80)
5.53
Emricasan, 2020
0.10 (0.07, 0.15)
5.63
Firsocostat, 2021
0.29 (0.17, 0.46)
4.89
Lanifibranor, 2020
0.45 (0.38, 0.53)
5.47
Liraglutide, 2016
0.43 (0.26, 0.63)
4.42
MSDC-0602k, 2020
0.34 (0.29, 0.40)
5.56
Obeticholic acid, 2014
0.45 (0.36, 0.55)
5.36
Obeticholic acid, 2019
0.33 (0.30, 0.37)
5.64
Pioglitazone, 2010
0.34 (0.24, 0.45)
5.29
Pioglitazone, 2016
0.48 (0.36, 0.61)
5.12
Resmetirom, 2019
0.56 (0.45, 0.67)
5.22
Selonsertib, 2018
0.21 (0.12, 0.33)
5.29
Simtuzumab (F3), 2018
0.22 (0.15, 0.30)
5.50
Simtuzumab (F4), 2018
0.18 (0.12, 0.25)
5.52
Volixibat, 2020
0.30 (0.17, 0.48)
4.79
Overall (I^2 = 96.10%, p = 0.00)
0.37 (0.28, 0.46)
100.00
0
0.2
0.4
0.6
0.8
1
