# Supplementary material for: Systematic review and meta-analysis: analysis of variables influencing the interpretation of clinical trial results in NAFLD
Source: J Gastroenterol. 2022 Mar 24;57(5):357–71. doi: 10.1007/s00535-022-01860-0 (PMC9016009; doi:10.1007/s00535-022-01860-0)
Supplement: Supplementary file 9 — Supplementary file9 (PPTX 55 KB) [file 535_2022_1860_MOESM9_ESM.pptx]

## Slide 1
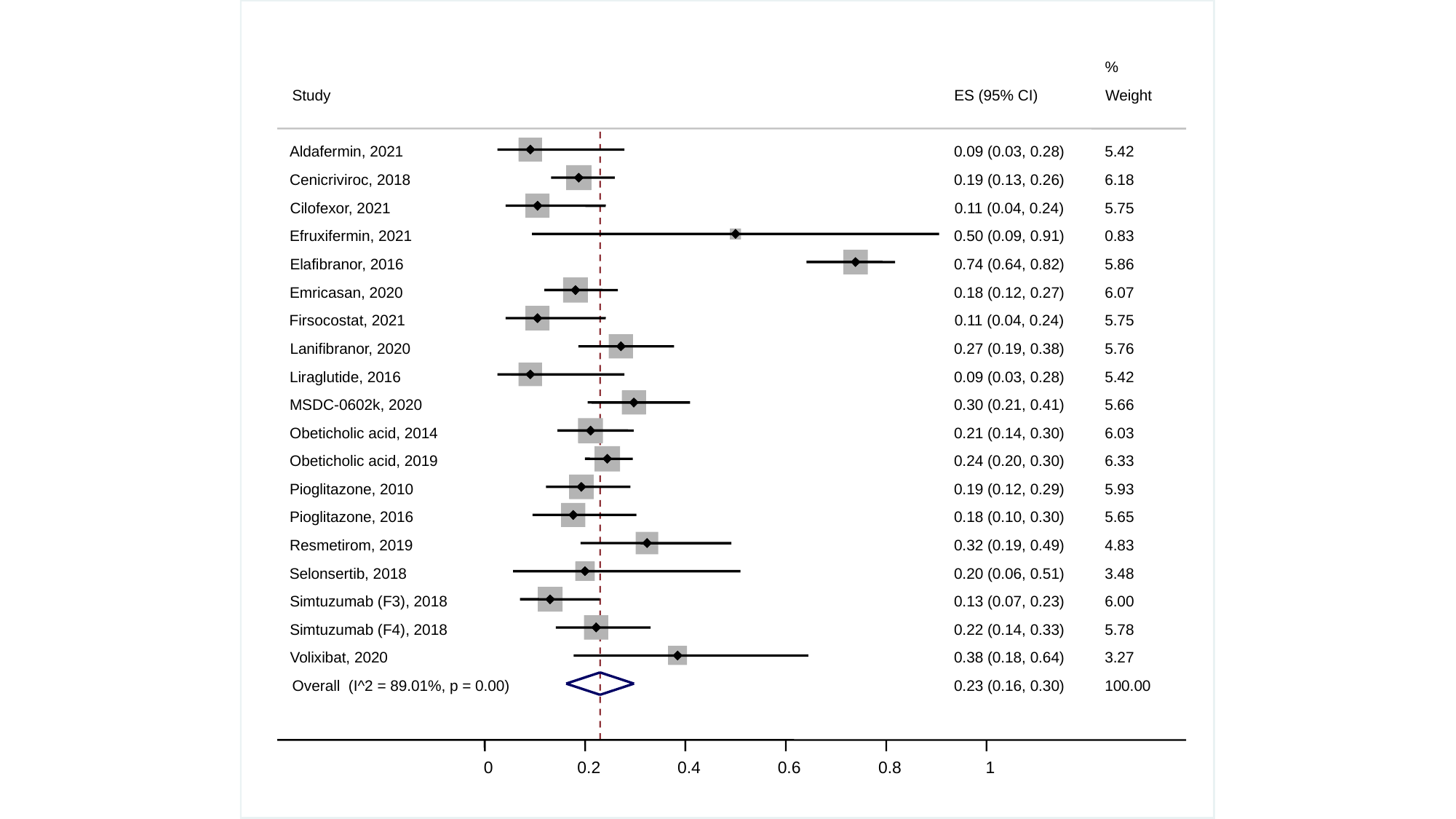

%
Study
ES (95% CI)
Weight
Aldafermin, 2021
0.09 (0.03, 0.28)
5.42
Cenicriviroc, 2018
0.19 (0.13, 0.26)
6.18
Cilofexor, 2021
0.11 (0.04, 0.24)
5.75
Efruxifermin, 2021
0.50 (0.09, 0.91)
0.83
Elafibranor, 2016
0.74 (0.64, 0.82)
5.86
Emricasan, 2020
0.18 (0.12, 0.27)
6.07
Firsocostat, 2021
0.11 (0.04, 0.24)
5.75
Lanifibranor, 2020
0.27 (0.19, 0.38)
5.76
Liraglutide, 2016
0.09 (0.03, 0.28)
5.42
MSDC-0602k, 2020
0.30 (0.21, 0.41)
5.66
Obeticholic acid, 2014
0.21 (0.14, 0.30)
6.03
Obeticholic acid, 2019
0.24 (0.20, 0.30)
6.33
Pioglitazone, 2010
0.19 (0.12, 0.29)
5.93
Pioglitazone, 2016
0.18 (0.10, 0.30)
5.65
Resmetirom, 2019
0.32 (0.19, 0.49)
4.83
Selonsertib, 2018
0.20 (0.06, 0.51)
3.48
Simtuzumab (F3), 2018
0.13 (0.07, 0.23)
6.00
Simtuzumab (F4), 2018
0.22 (0.14, 0.33)
5.78
Volixibat, 2020
0.38 (0.18, 0.64)
3.27
Overall (I^2 = 89.01%, p = 0.00)
0.23 (0.16, 0.30)
100.00
0
0.2
0.4
0.6
0.8
1
