# Supplementary material for: Systematic review and meta-analysis: analysis of variables influencing the interpretation of clinical trial results in NAFLD
Source: J Gastroenterol. 2022 Mar 24;57(5):357–71. doi: 10.1007/s00535-022-01860-0 (PMC9016009; doi:10.1007/s00535-022-01860-0)
Supplement: Supplementary file 13 — Supplementary file13 (PPTX 61 KB) [file 535_2022_1860_MOESM13_ESM.pptx]

## Slide 1
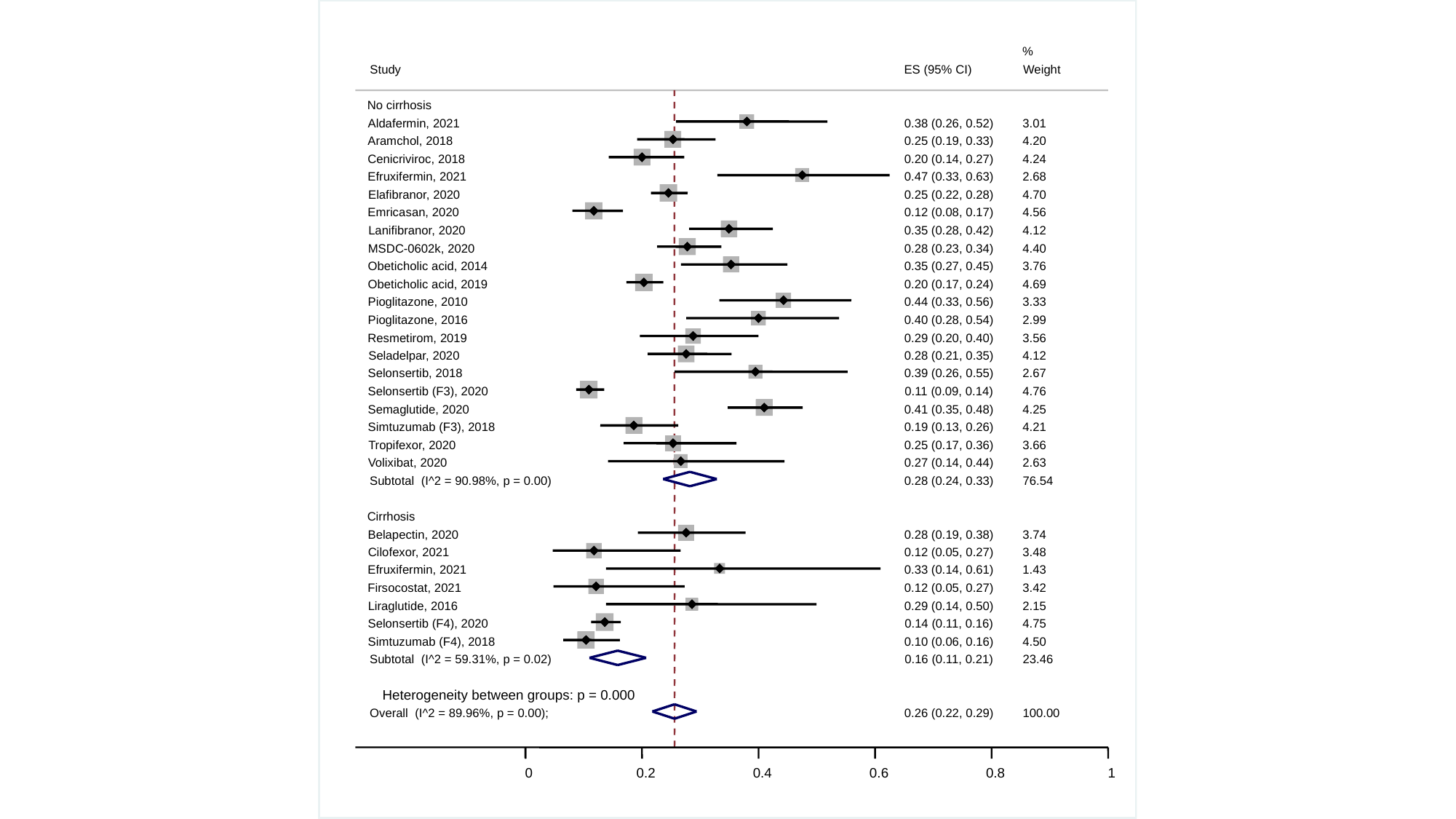

Study
ES (95% CI)
No cirrhosis
Aldafermin, 2021
Aramchol, 2018
Cenicriviroc, 2018
Efruxifermin, 2021
Elafibranor, 2020
Emricasan, 2020
0.12 (0.08, 0.17)
Lanifibranor, 2020
MSDC-0602k, 2020
Obeticholic acid, 2014
Obeticholic acid, 2019
Pioglitazone, 2010
0.44 (0.33, 0.56)
Pioglitazone, 2016
0.40 (0.28, 0.54)
Resmetirom, 2019
Seladelpar, 2020
0.28 (0.21, 0.35)
Selonsertib, 2018
Selonsertib (F3), 2020
Semaglutide, 2020
0.41 (0.35, 0.48)
Simtuzumab (F3), 2018
0.19 (0.13, 0.26)
Tropifexor, 2020
0.25 (0.17, 0.36)
Volixibat, 2020
Subtotal (I^2 = 90.98%, p = 0.00)
0.28 (0.24, 0.33)
Cirrhosis
Belapectin, 2020
0.28 (0.19, 0.38)
Cilofexor, 2021
Efruxifermin, 2021
0.33 (0.14, 0.61)
Firsocostat, 2021
Liraglutide, 2016
0.29 (0.14, 0.50)
Selonsertib (F4), 2020
Simtuzumab (F4), 2018
Subtotal (I^2 = 59.31%, p = 0.02)
Heterogeneity between groups: p = 0.000
Overall (I^2 = 89.96%, p = 0.00);
0.26 (0.22, 0.29)
%
Weight
0.38 (0.26, 0.52)
3.01
0.25 (0.19, 0.33)
4.20
0.20 (0.14, 0.27)
4.24
0.47 (0.33, 0.63)
2.68
0.25 (0.22, 0.28)
4.70
4.56
0.35 (0.28, 0.42)
4.12
0.28 (0.23, 0.34)
4.40
0.35 (0.27, 0.45)
3.76
0.20 (0.17, 0.24)
4.69
3.33
2.99
0.29 (0.20, 0.40)
3.56
4.12
0.39 (0.26, 0.55)
2.67
0.11 (0.09, 0.14)
4.76
4.25
4.21
3.66
0.27 (0.14, 0.44)
2.63
76.54
3.74
0.12 (0.05, 0.27)
3.48
1.43
0.12 (0.05, 0.27)
3.42
2.15
0.14 (0.11, 0.16)
4.75
0.10 (0.06, 0.16)
4.50
0.16 (0.11, 0.21)
23.46
100.00
0
0.2
0.4
0.6
0.8
1
