# Supplementary material for: Systematic review and meta-analysis: analysis of variables influencing the interpretation of clinical trial results in NAFLD
Source: J Gastroenterol. 2022 Mar 24;57(5):357–71. doi: 10.1007/s00535-022-01860-0 (PMC9016009; doi:10.1007/s00535-022-01860-0)
Supplement: Supplementary file 14 — Supplementary file14 (PPTX 61 KB) [file 535_2022_1860_MOESM14_ESM.pptx]

## Slide 1
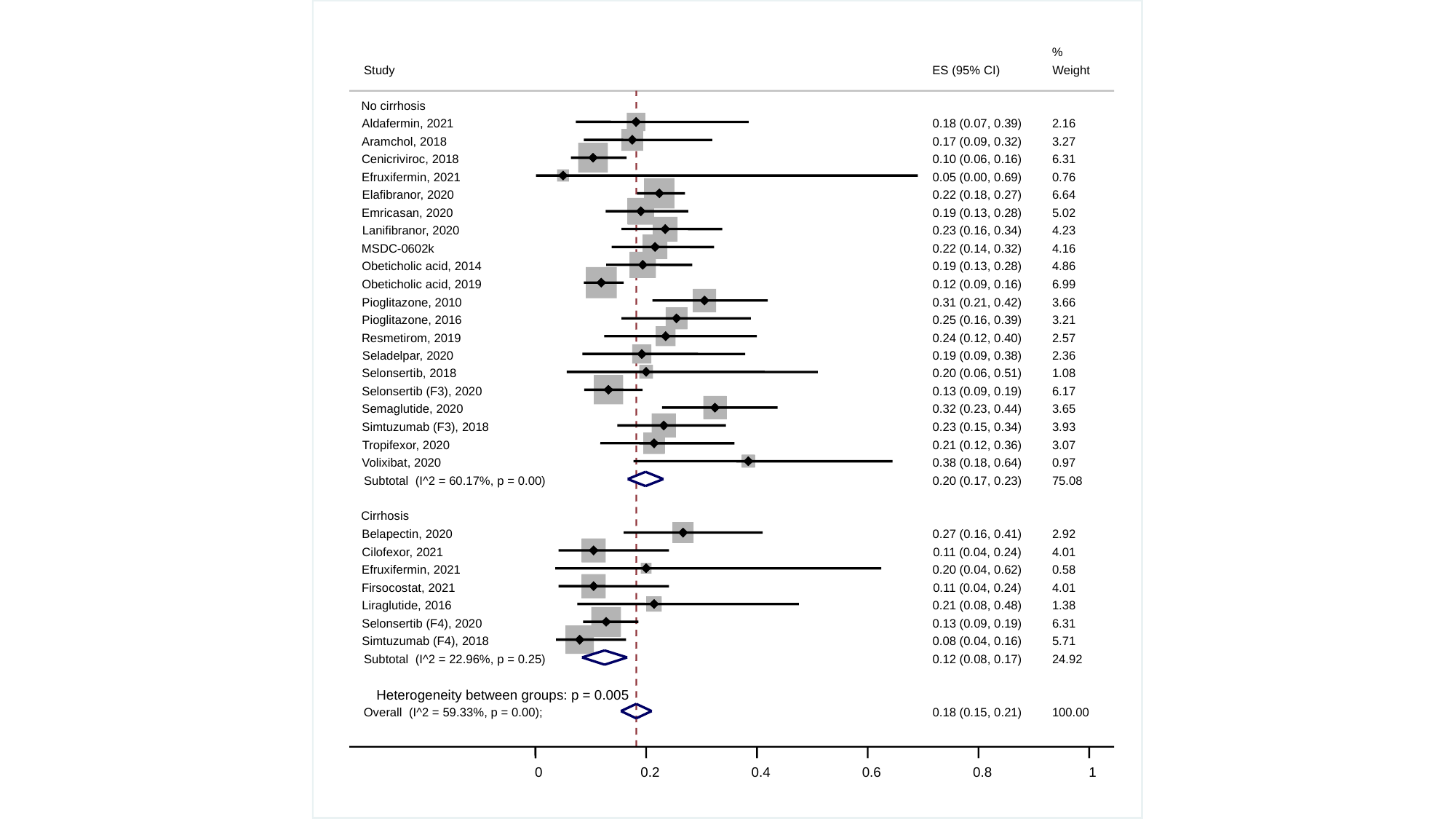

Study
No cirrhosis
Aldafermin, 2021
Aramchol, 2018
Cenicriviroc, 2018
Efruxifermin, 2021
0.05 (0.00, 0.69)
Elafibranor, 2020
0.22 (0.18, 0.27)
Emricasan, 2020
Lanifibranor, 2020
0.23 (0.16, 0.34)
MSDC-0602k
Obeticholic acid, 2014
0.19 (0.13, 0.28)
Obeticholic acid, 2019
Pioglitazone, 2010
0.31 (0.21, 0.42)
Pioglitazone, 2016
Resmetirom, 2019
Seladelpar, 2020
0.19 (0.09, 0.38)
Selonsertib, 2018
0.20 (0.06, 0.51)
Selonsertib (F3), 2020
Semaglutide, 2020
Simtuzumab (F3), 2018
Tropifexor, 2020
0.21 (0.12, 0.36)
Volixibat, 2020
Subtotal (I^2 = 60.17%, p = 0.00)
Cirrhosis
Belapectin, 2020
0.27 (0.16, 0.41)
Cilofexor, 2021
Efruxifermin, 2021
Firsocostat, 2021
0.11 (0.04, 0.24)
Liraglutide, 2016
Selonsertib (F4), 2020
0.13 (0.09, 0.19)
Simtuzumab (F4), 2018
Subtotal (I^2 = 22.96%, p = 0.25)
0.12 (0.08, 0.17)
Heterogeneity between groups: p = 0.005
Overall (I^2 = 59.33%, p = 0.00);
0.18 (0.15, 0.21)
%
ES (95% CI)
Weight
0.18 (0.07, 0.39)
2.16
0.17 (0.09, 0.32)
3.27
0.10 (0.06, 0.16)
6.31
0.76
6.64
0.19 (0.13, 0.28)
5.02
4.23
0.22 (0.14, 0.32)
4.16
4.86
0.12 (0.09, 0.16)
6.99
3.66
0.25 (0.16, 0.39)
3.21
0.24 (0.12, 0.40)
2.57
2.36
1.08
0.13 (0.09, 0.19)
6.17
0.32 (0.23, 0.44)
3.65
0.23 (0.15, 0.34)
3.93
3.07
0.38 (0.18, 0.64)
0.97
0.20 (0.17, 0.23)
75.08
2.92
0.11 (0.04, 0.24)
4.01
0.20 (0.04, 0.62)
0.58
4.01
0.21 (0.08, 0.48)
1.38
6.31
0.08 (0.04, 0.16)
5.71
24.92
100.00
0
0.2
0.4
0.6
0.8
1
