# Supplementary material for: Systematic review and meta-analysis: analysis of variables influencing the interpretation of clinical trial results in NAFLD
Source: J Gastroenterol. 2022 Mar 24;57(5):357–71. doi: 10.1007/s00535-022-01860-0 (PMC9016009; doi:10.1007/s00535-022-01860-0)
Supplement: Supplementary file 15 — Supplementary file15 (PPTX 56 KB) [file 535_2022_1860_MOESM15_ESM.pptx]

## Slide 1
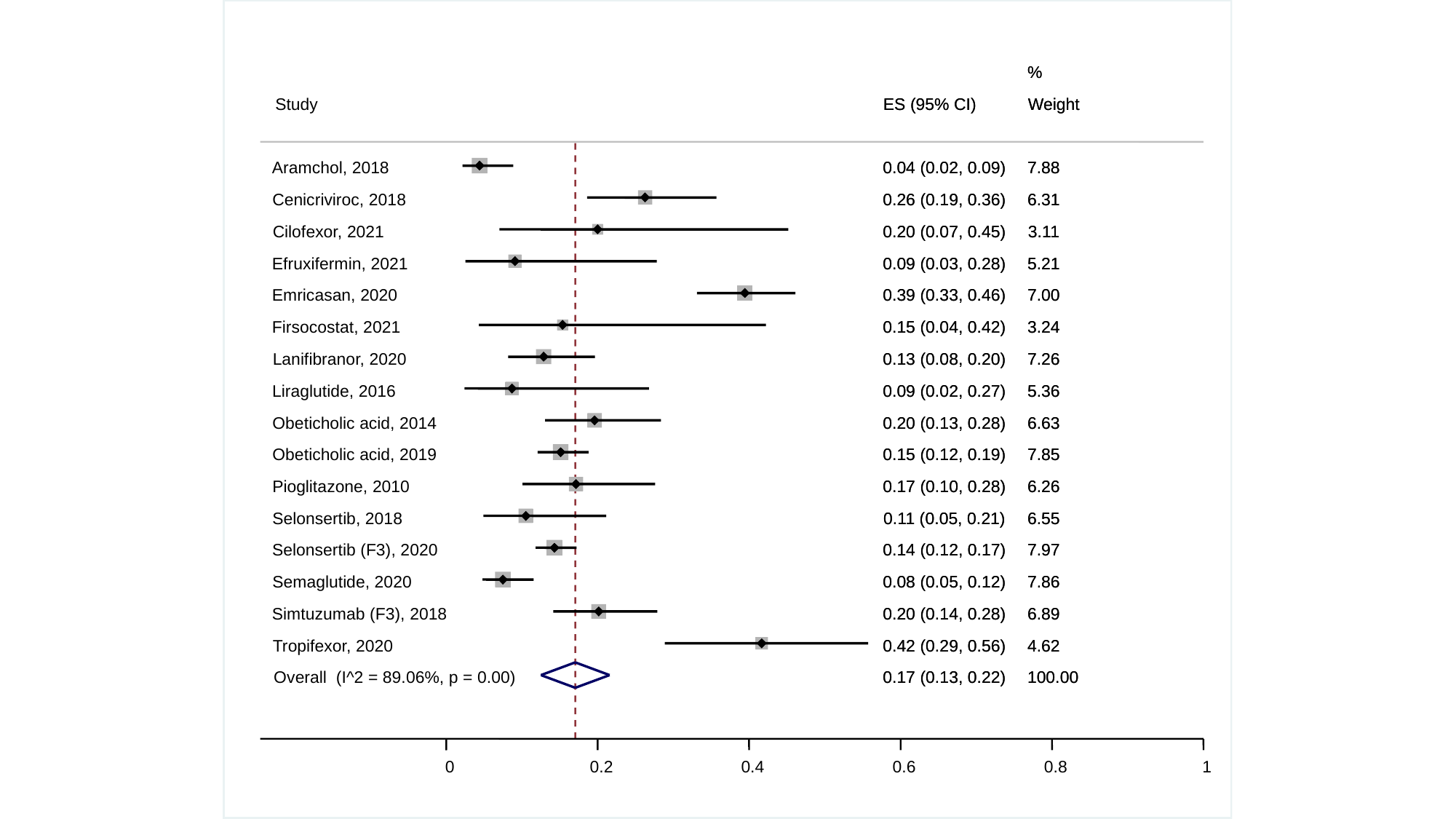

%
%
Study
ES (95% CI)
ES (95% CI)
Weight
Weight
Aramchol, 2018
0.04 (0.02, 0.09)
0.04 (0.02, 0.09)
7.88
7.88
Cenicriviroc, 2018
0.26 (0.19, 0.36)
0.26 (0.19, 0.36)
6.31
6.31
Cilofexor, 2021
0.20 (0.07, 0.45)
0.20 (0.07, 0.45)
3.11
3.11
Efruxifermin, 2021
0.09 (0.03, 0.28)
0.09 (0.03, 0.28)
5.21
5.21
Emricasan, 2020
0.39 (0.33, 0.46)
0.39 (0.33, 0.46)
7.00
7.00
Firsocostat, 2021
0.15 (0.04, 0.42)
0.15 (0.04, 0.42)
3.24
3.24
Lanifibranor, 2020
0.13 (0.08, 0.20)
0.13 (0.08, 0.20)
7.26
7.26
Liraglutide, 2016
0.09 (0.02, 0.27)
0.09 (0.02, 0.27)
5.36
5.36
Obeticholic acid, 2014
0.20 (0.13, 0.28)
0.20 (0.13, 0.28)
6.63
6.63
Obeticholic acid, 2019
0.15 (0.12, 0.19)
0.15 (0.12, 0.19)
7.85
7.85
Pioglitazone, 2010
0.17 (0.10, 0.28)
0.17 (0.10, 0.28)
6.26
6.26
Selonsertib, 2018
0.11 (0.05, 0.21)
0.11 (0.05, 0.21)
6.55
6.55
Selonsertib (F3), 2020
0.14 (0.12, 0.17)
0.14 (0.12, 0.17)
7.97
7.97
Semaglutide, 2020
0.08 (0.05, 0.12)
0.08 (0.05, 0.12)
7.86
7.86
Simtuzumab (F3), 2018
0.20 (0.14, 0.28)
0.20 (0.14, 0.28)
6.89
6.89
Tropifexor, 2020
0.42 (0.29, 0.56)
0.42 (0.29, 0.56)
4.62
4.62
Overall (I^2 = 89.06%, p = 0.00)
0.17 (0.13, 0.22)
0.17 (0.13, 0.22)
100.00
100.00
0
0.2
0.4
0.6
0.8
1
