# Supplementary material for: Systematic review and meta-analysis: analysis of variables influencing the interpretation of clinical trial results in NAFLD
Source: J Gastroenterol. 2022 Mar 24;57(5):357–71. doi: 10.1007/s00535-022-01860-0 (PMC9016009; doi:10.1007/s00535-022-01860-0)
Supplement: Supplementary file 16 — Supplementary file16 (PPTX 57 KB) [file 535_2022_1860_MOESM16_ESM.pptx]

## Slide 1
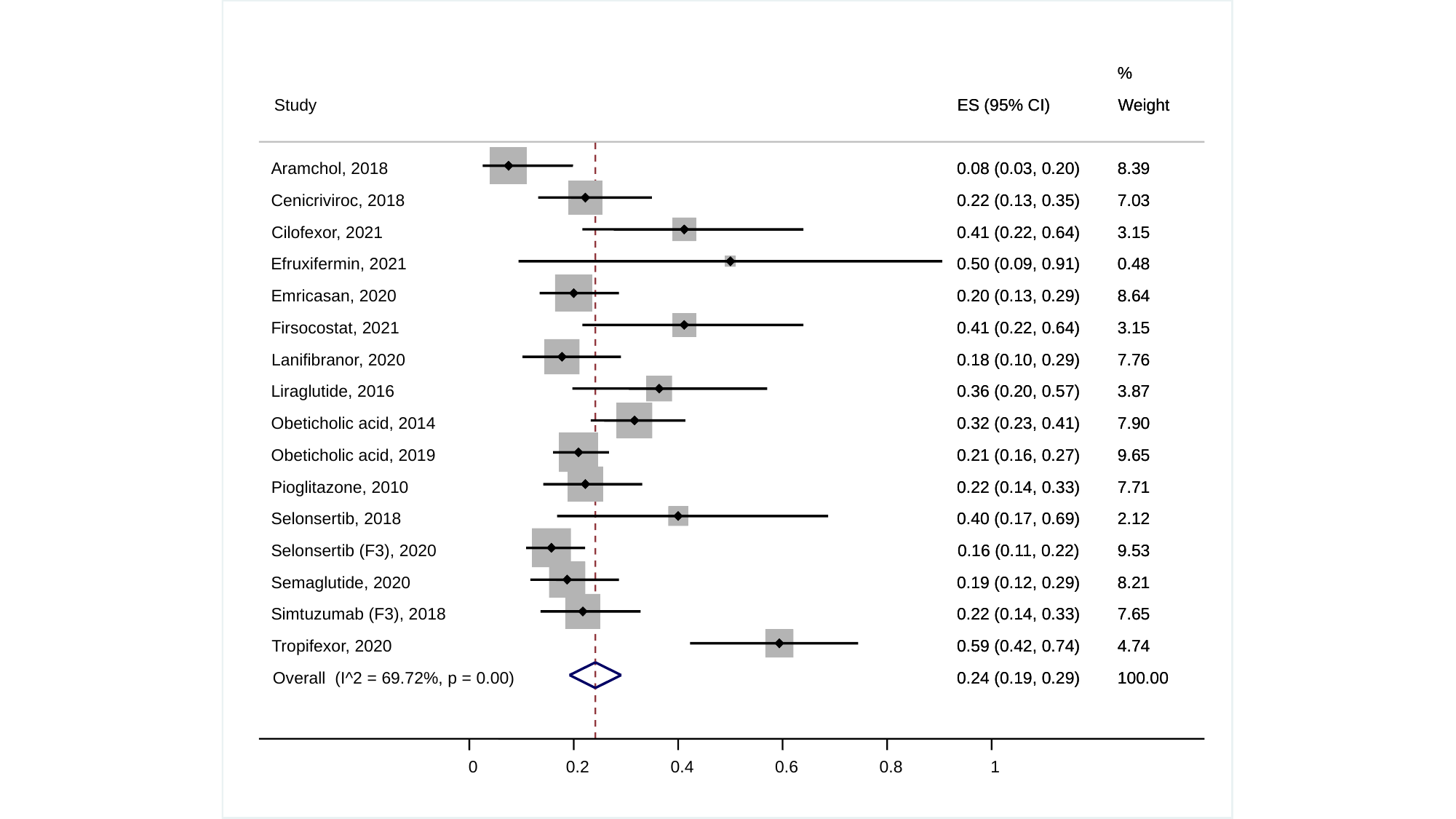

%
%
Study
ES (95% CI)
ES (95% CI)
Weight
Weight
Aramchol, 2018
0.08 (0.03, 0.20)
0.08 (0.03, 0.20)
8.39
8.39
Cenicriviroc, 2018
0.22 (0.13, 0.35)
0.22 (0.13, 0.35)
7.03
7.03
Cilofexor, 2021
0.41 (0.22, 0.64)
0.41 (0.22, 0.64)
3.15
3.15
Efruxifermin, 2021
0.50 (0.09, 0.91)
0.50 (0.09, 0.91)
0.48
0.48
Emricasan, 2020
0.20 (0.13, 0.29)
0.20 (0.13, 0.29)
8.64
8.64
Firsocostat, 2021
0.41 (0.22, 0.64)
0.41 (0.22, 0.64)
3.15
3.15
Lanifibranor, 2020
0.18 (0.10, 0.29)
0.18 (0.10, 0.29)
7.76
7.76
Liraglutide, 2016
0.36 (0.20, 0.57)
0.36 (0.20, 0.57)
3.87
3.87
Obeticholic acid, 2014
0.32 (0.23, 0.41)
0.32 (0.23, 0.41)
7.90
7.90
Obeticholic acid, 2019
0.21 (0.16, 0.27)
0.21 (0.16, 0.27)
9.65
9.65
Pioglitazone, 2010
0.22 (0.14, 0.33)
0.22 (0.14, 0.33)
7.71
7.71
Selonsertib, 2018
0.40 (0.17, 0.69)
0.40 (0.17, 0.69)
2.12
2.12
Selonsertib (F3), 2020
0.16 (0.11, 0.22)
0.16 (0.11, 0.22)
9.53
9.53
Semaglutide, 2020
0.19 (0.12, 0.29)
0.19 (0.12, 0.29)
8.21
8.21
Simtuzumab (F3), 2018
0.22 (0.14, 0.33)
0.22 (0.14, 0.33)
7.65
7.65
Tropifexor, 2020
0.59 (0.42, 0.74)
0.59 (0.42, 0.74)
4.74
4.74
Overall (I^2 = 69.72%, p = 0.00)
0.24 (0.19, 0.29)
0.24 (0.19, 0.29)
100.00
100.00
0
0.2
0.4
0.6
0.8
1
