# Supplementary material for: Systematic review and meta-analysis: analysis of variables influencing the interpretation of clinical trial results in NAFLD
Source: J Gastroenterol. 2022 Mar 24;57(5):357–71. doi: 10.1007/s00535-022-01860-0 (PMC9016009; doi:10.1007/s00535-022-01860-0)
Supplement: Supplementary file 18 — Supplementary file18 (DOCX 13 KB) [file 535_2022_1860_MOESM18_ESM.docx]

| **Antifibrotic** | **Antipoptotic** | **Antimetabolic** | **DNL** | **FGF analogues** | **FXR agonists** |
| --- | --- | --- | --- | --- | --- |
| Belapectin | Emricasan | Elafibranor | Aramchol | Aldafermin | Obeticholic Acid |
| Cenicriviroc |  | Lanifibranor | Firsocostat | Efruxifermin | Cilofexor |
| Selonsertib |  | Liraglutide | Resmetirom |  | Tropifexor |
| Simtuzumab |  | MSDC-0602k |  |  |  |
|  |  | Pioglitazone |  |  |  |
|  |  | Seladelpar |  |  |  |
|  |  | Semaglutide |  |  |  |
|  |  | Volixibat |  |  |  |
