# Supplementary material for: Systematic review and meta-analysis: analysis of variables influencing the interpretation of clinical trial results in NAFLD
Source: J Gastroenterol. 2022 Mar 24;57(5):357–71. doi: 10.1007/s00535-022-01860-0 (PMC9016009; doi:10.1007/s00535-022-01860-0)
Supplement: Supplementary file 19 — Supplementary file19 (DOCX 20 KB) [file 535_2022_1860_MOESM19_ESM.docx]

| **Drug** | **Year** | **Study population** | **Study Attrition** | **Prognostic factor measurement** | **Outcome measurement** | **Confounding assessment & account** | **Data analysis & reporting** |
| --- | --- | --- | --- | --- | --- | --- | --- |
| Aldafermin | 2021 | Low | Low | Low | Low | Low | Low |
| Aramchol | 2020 | Low | N/A | Low | Low | N/A | Low |
| Belapectin | 2020 | Low | Moderate | Low | Low | Low | Low |
| Cenicriviroc | 2017 | Low | Moderate | Low | Low | Low | Low |
| Cilofexor-Fircostotat | 2020 | Low | Low | Low | Low | Low | Low |
| Efruxifermin | 2021 | Low | Low | Low | Low | Low | Low |
| Efruxifermin | 2021 | N/A | N/A | N/A | N/A | N/A | N/A |
| Elafibranor | 2016 | Low | Moderate | Low | Low | Low | Low |
| Elafibranor | 2020 | Low | Moderate | Low | Low | N/A | Low |
| Emricasan | 2020 | Low | High | Low | Low | Low | Low |
| Lanifibranor | 2021 | Low | Low | Low | Low | Low | Low |
| Liraglutide | 2016 | Low | Moderate | Low | Low | Low | Low |
| MSDC-0602K | 2019 | Low | Moderate | Low | Low | Low | Low |
| Obeticholic Acid | 2014 | Low | Low | Low | Low | Low | Low |
| Obeticholic acid | 2019 | Low | Low | Low | Low | Moderate | Low |
| Pioglitazone | 2010 | Low | Low | Low | Low | Low | Low |
| Pioglitazone | 2016 | Low | Low | Low | Low | Low | Low |
| Resmetirom | 2019 | Low | Low | Low | Low | Low | Low |
| Seladelpar | 2020 | Low | Moderate | Low | Low | N/A | Low |
| Selonsertib | 2017 | Low | Moderate | Low | Low | High | Low |
| Selonsertib | 2020 | Low | Moderate | Low | Low | High | Low |
| Semaglutide | 2021 | Low | Moderate | Low | Low | Low | Low |
| Simtuzumab | 2018 | Low | Moderate | Low | Low | High | Low |
| Tropifexor | 2020 | Low | Moderate | Low | Low | N/A | Low |
| Volixibat | 2020 | Low | Moderate | Low | Low | High | Low |

N/A: Not available.
