# Supplementary material for: Systematic review and meta-analysis: analysis of variables influencing the interpretation of clinical trial results in NAFLD
Source: J Gastroenterol. 2022 Mar 24;57(5):357–71. doi: 10.1007/s00535-022-01860-0 (PMC9016009; doi:10.1007/s00535-022-01860-0)
Supplement: Supplementary file 20 — Supplementary file20 (DOCX 14 KB) [file 535_2022_1860_MOESM20_ESM.docx]

| **Study omitted** | **Estimate [95% Conf. Interval]** |
| --- | --- |
| Aldafermin 2021 | 1.6304815 1.2360345 2.1508055 |
| Aramchol 2018 | 1.6549834 1.2498755 2.1913943 |
| Cenicriviroc 2018 | 1.6695671 1.2571237 2.2173271 |
| Cilofexor 2021 | 1.6539733 1.2598161 2.1714504 |
| Efruxifermin 2021 | 1.6637653 1.2625432 2.1924913 |
| Efruxifermin 2021 | 1.6444662 1.2488827 2.1653507 |
| Elafibranor 2016 | 1.6805034 1.2590173 2.2430921 |
| Elafibranor 2020 | 1.6878239 1.2474523 2.2836542 |
| Emricasan 2020 | 1.7513298 1.3484361 2.2746024 |
| Firsocostat 2020 | 1.6469203 1.2504038 2.1691766 |
| Lanifibranor 2021 | 1.599771 1.2082038 2.1182415 |
| Liraglutide 2016 | 1.6066434 1.2251586 2.1069136 |
| MSDC-0602k 2020 | 1.6742944 1.2533648 2.2365887 |
| Obeticholic acid 2014 | 1.6494089 1.2382294 2.197129 |
| Obeticholic acid 2019 | 1.6742803 1.2459016 2.2499487 |
| Pioglitazone 2010 | 1.5837317 1.2030433 2.0848842 |
| Pioglitazone 2016 | 1.5719842 1.2011062 2.0573821 |
| Resmetirom 2019 | 1.6118084 1.2259911 2.1190419 |
| Seladelpar 2020 | 1.6334811 1.2372248 2.1566496 |
| Selonsertib 2020 | 1.7719966 1.3791788 2.2766967 |
| Selonsertib 2020 | 1.7215941 1.3068384 2.2679822 |
| Semaglutide 2020 | 1.5668627 1.1958711 2.0529459 |
| Simtuzumab 2018 | 1.6463362 1.2460203 2.1752639 |
| Simtuzumab 2018 | 1.6728048 1.2650359 2.212013 |
| Tropifexor 2020 | 1.6465473 1.2445265 2.1784334 |
| Volixibat 2020 | 1.7079397 1.30097 2.2422178 |
