# Supplementary material for: Systematic review and meta-analysis: analysis of variables influencing the interpretation of clinical trial results in NAFLD
Source: J Gastroenterol. 2022 Mar 24;57(5):357–71. doi: 10.1007/s00535-022-01860-0 (PMC9016009; doi:10.1007/s00535-022-01860-0)
Supplement: Supplementary file 21 — Supplementary file21 (DOCX 14 KB) [file 535_2022_1860_MOESM21_ESM.docx]

| **Study omitted** | **Estimate [95% Conf. Interval]** |
| --- | --- |
| Aldafermin 2021 | 1.3153284 1.132722 1.5273727 |
| Aramchol 2018 | 1.3248984 1.133935 1.5480213 |
| Belapectin 2020 | 1.3418179 1.1484997 1.5676756 |
| Cenicriviroc 2018 | 1.2997168 1.1198529 1.5084693 |
| Cilofexor 2021 | 1.3339674 1.1428676 1.5570214 |
| Efruxifermin 2021 | 1.3267438 1.1400608 1.543996 |
| Efruxifermin 2021 | 1.3295027 1.1400499 1.5504385 |
| Elafibranor 2020 | 1.3658594 1.1601744 1.6080101 |
| Emricasan 2020 | 1.3710274 1.1965388 1.570961 |
| Firsocostat 2020 | 1.3335128 1.1424074 1.556587 |
| Lanifibranor 2021 | 1.3105847 1.1220975 1.5307333 |
| Liraglutide 2016 | 1.3305399 1.1399803 1.5529535 |
| MSDC-0602k 2020 | 1.3288182 1.1338593 1.5572988 |
| Obeticholic acid 2014 | 1.2927866 1.1174593 1.4956225 |
| Obeticholic acid 2019 | 1.2675149 1.0939145 1.4686651 |
| Pioglitazone 2010 | 1.3129318 1.1249764 1.5322899 |
| Pioglitazone 2016 | 1.3154016 1.1285273 1.5332205 |
| Resmetirom 2019 | 1.3323479 1.139769 1.5574654 |
| Seladelpar 2020 | 1.3265766 1.1358936 1.5492697 |
| Selonsertib 2018 | 1.3234897 1.1367966 1.5408429 |
| Selonsertib 2020 | 1.3704287 1.1882192 1.5805795 |
| Selonsertib 2020 | 1.3529569 1.1551458 1.5846418 |
| Semaglutide 2020 | 1.325001 1.1298269 1.5538909 |
| Simtuzumab 2018 | 1.3564857 1.1709329 1.5714424 |
| Simtuzumab 2018 | 1.3318104 1.1395164 1.5565542 |
| Tropifexor 2020 | 1.334303 1.1413013 1.5599426 |
| Volixibat 2020 | 1.3417422 1.1555507 1.557934 |
