# Supplementary material for: Systematic review and meta-analysis: analysis of variables influencing the interpretation of clinical trial results in NAFLD
Source: J Gastroenterol. 2022 Mar 24;57(5):357–71. doi: 10.1007/s00535-022-01860-0 (PMC9016009; doi:10.1007/s00535-022-01860-0)
Supplement: Supplementary file 22 — Supplementary file22 (DOCX 14 KB) [file 535_2022_1860_MOESM22_ESM.docx]

| **Study omitted** | **Estimate [95% Conf. Interval]** |
| --- | --- |
| Aramchol 2018 | .65339452 .45651823 .93517494 |
| Cenicriviroc 2018 | .61711913 .42890131 .88793391 |
| Cilofexor 2021 | .66572636 .46751994 .94796294 |
| Efruxifermin 2021 | .66793352 .47330916 .94258738 |
| Emricasan 2020 | .62238103 .48940155 .79149348 |
| Firsocostat 2020 | .67099351 .47301206 .951841 |
| Lanifibranor 2021 | .64434522 .44515806 .93265915 |
| Liraglutide 2016 | .68587136 .48715144 .96565366 |
| Obeticholic acid 2014 | .66034997 .45576319 .95677328 |
| Obeticholic acid 2019 | .63487321 .42818931 .94132191 |
| Pioglitazone 2010 | .64158785 .44323504 .92870581 |
| Selonsertib 2018 | .69051421 .49103385 .97103256 |
| Selonsertib 2020 | .61967224 .4209328 .91224468 |
| Semaglutide 2020 | .69313878 .48809025 .98432893 |
| Simtuzumab 2018 | .62772983 .43228975 .91152912 |
| Tropifexor 2020 | .6631003 .46114904 .95349222 |
