# Supplementary material for: Systematic review and meta-analysis: analysis of variables influencing the interpretation of clinical trial results in NAFLD
Source: J Gastroenterol. 2022 Mar 24;57(5):357–71. doi: 10.1007/s00535-022-01860-0 (PMC9016009; doi:10.1007/s00535-022-01860-0)
Supplement: Supplementary file 23 — Supplementary file23 (DOCX 13 KB) [file 535_2022_1860_MOESM23_ESM.docx]

|  | **NASH resolution** | **Fibrosis improvement** | **Fibrosis progression** |
| --- | --- | --- | --- |
| Cirrhosis (%) | 0.520 | 0.464 | 0.644 |
| NAS (mean value) | 0.009 | 0.673 | 0.373 |
| T2DM (%) | 0.233 | 0.256 | 0.900 |
| BMI (mean value) | 0.344 | 0.702 | 0.114 |
| Length (weeks) | 0.675 | 0.317 | 0.170 |
| Therapeutic class | 0.626 | 0.010 | 0.509 |
| Sample size (number of patients) | 0.578 | 0.565 | 0.683 |
